# A dimerization-dependent mechanism regulates enzymatic activation and nuclear entry of PLK1

**DOI:** 10.1038/s41388-021-02094-9

**Published:** 2021-11-10

**Authors:** Monika Raab, Yves Matthess, Christopher A. Raab, Niklas Gutfreund, Volker Dötsch, Sven Becker, Mourad Sanhaji, Klaus Strebhardt

**Affiliations:** 1 Department of Gynecology, Medical School, Goethe University, Frankfurt, Germany; 2 Institute of Biophysical Chemistry and Center for Biomolecular Magnetic Resonance, Goethe University, Max-von-Laue Str. 9, 60438 Frankfurt am Main, Germany; 3 German Cancer Consortium (DKTK) / German Cancer Research Center, Heidelberg, Germany

**Keywords:** Mitosis, Cell division

## Abstract

Polo-like kinase 1 (PLK1) is a crucial regulator of cell cycle progression. It is established that the activation of PLK1 depends on the coordinated action of Aurora-A and Bora. Nevertheless, very little is known about the spatiotemporal regulation of PLK1 during G2, specifically, the mechanisms that keep cytoplasmic PLK1 inactive until shortly before mitosis onset. Here, we describe PLK1 dimerization as a new mechanism that controls PLK1 activation. During the early G2 phase, Bora supports transient PLK1 dimerization, thus fine-tuning the timely regulated activation of PLK1 and modulating its nuclear entry. At late G2, the phosphorylation of T210 by Aurora-A triggers dimer dissociation and generates active PLK1 monomers that support entry into mitosis. Interfering with this critical PLK1 dimer/monomer switch prevents the association of PLK1 with importins, limiting its nuclear shuttling, and causes nuclear PLK1 mislocalization during the G2-M transition. Our results suggest a novel conformational space for the design of a new generation of PLK1 inhibitors.

## Introduction

PLK1 is a serine/threonine kinase that plays a pivotal role in cell division [1–6]. Its dysregulation is tightly linked to malignant transformation [7, 8]. In human cancers, there is growing evidence coupling PLK1 activity to tumor development, progression, and therapy resistance [9–12]. PLK1 is overexpressed in various cancers, and its expression levels correlate with poor prognosis [13–15]. During cell division, the most prominent network orchestrating mitotic onset consists of the PLK1-Aurora A-Cyclin B1/CDK1 feedback loop. In this respect, PLK1 activity is reported to be a critical prerequisite for mitotic entry.

Through phosphorylation of CDC25C1, WEE1, and MYT1, PLK1 promotes the activation of the mitotic driver Cyclin B1/CDK1 in the late G2-phase triggering prophase onset [16–18]. During the G2 phase and mitosis, different pools of PLK1 are involved in centrosome maturation, kinetochore functions, chromosome condensation, spindle assembly, and cytokinesis [19–21]. While the N-terminal domain of PLK1 encompasses the kinase domain (KD), the C-terminal domain contains the Polo-Box domain (PBD), which promotes the recruitment of PLK1 to different subcellular structures and contributes to the regulation of PLK1 activity [3, 6, 22, 23]. Regarding PLK1 activation, a potential model has been described in Danio rerio, which involves the intramolecular interaction between the KD and PBD [24]. During interphase, the KD, the PBD, and the interdomain linker engage in an allosteric inhibitory interaction keeping the kinase in an inactive state. During mitosis, the PBD, a phosphoserine/threonine recognition domain, binds to pre-phosphorylated proteins, thus abrogating the inhibitory interaction between KD and PBD. As a consequence, the T-loop region of PLK1 encompassing threonine 210 (T210) becomes accessible for phosphorylation by the upstream kinase Aurora A (Aur-A), in concert with the co-factor Bora, achieving full activation of PLK1 [23, 25, 26]. Initial studies proposed that Bora promotes the activation of PLK1 at centrosomes and in the cytoplasm [27]. However, accumulating evidence suggests that activation of PLK1 at centrosomes involves the centrosome protein CEP192 and Aur-A [28, 29]. Thus, two distinct Aur-A-PLK1 cascades for PLK1 activation co-exist and work simultaneously at the G2 phase. The first is driven by CEP192 at centrosomes, whereas Bora, which is exclusively cytoplasmic, contributes to the activation of cytoplasmic PLK1 [30]. The fact that PLK1 activation occurs during G2 is well established [2, 30]. However, the initial activation and, in particular, where PLK1 is first activated remains questioned. Examples depicting divergences in published data come from two studies. On one side, Bruinsma et al. showed in an elegant experiment using a PLK1-regulated FRET-based biosensor (Myt1 phosphorylation sequence) the existence of a spatiotemporal separation of PLK1 activation and function [31]. Intriguingly, the authors found that although phosphorylated T210 is first observed at the centrosome, the substrate-directed activity of PLK1 is first spotted exclusively in the nucleus 5 h before mitosis. In contrast, the cytoplasmic activity of PLK1 was seen shortly before the nuclear envelop breakdown. On the other side, the second study conducted by Gheghiani et al. using an improved version of the FRET biosensor (c-Jun phosphorylation sequence), detected no significant changes in PLK1 activity during the early G2 phase but rather a sudden and rapid increase in activity in late G2, shortly before nuclear envelop breakdown. Interestingly, this activation surge has been observed simultaneously in the cytoplasm and the nucleus [16]. Still, the critical aspect raised in both studies is how the cytoplasmic pool of PLK1 is retained in an inhibited state during early G2 despite the local accumulation of the kinase activators, Aur-A and Bora. Here, we propose the PLK1 dimerization as a new mechanism taking part in activating PLK1 during the G2/M transition. During early G2, we confirmed the presence of PLK1 in complex with Aur-A and Bora. In this complex, Bora appears to play the role of a scaffold protein promoting the dimerization of PLK1 before Aur-A-dependent phosphorylation of T210, thereby contributing to the controlled activation of PLK1. During late G2 and concomitant to the increase T210 phosphorylation by Aur-A, the PLK1 dimer dissociates, allowing PLK1 to acquire full activity and to translocate into the nucleus. We found that blocking the dissociation of PLK1 dimers at the late G2 phase influenced PLK1´s cytoplasmic localization and, more importantly, interfered with the importin-dependent shuttling of PLK1 to the nucleus causing a reduced PLK1 threshold activity in mitotic cells.

## Materials and methods

### Cell culture and transfection

Cell lines were purchased from ATCC and cultivated according to the guidelines. DNA and siRNA transfections were performed using jetPEI DNA Transfection Reagent (Polyplus) and Lipofectamine RNAiMAX Transfection Reagent (Invitrogen), respectively according to the manufacturer’s instructions. Cell lysis was performed in RIPA buffer. The control siRNA (siC) and siBora were from Sigma.

### Tagging of endogenous Plk1 by CRISPR/CAS9

To target exon 10 of the Plk1 gene two protospacers were used (GCTCGGCCAGCAACCGTCTCA and GAGGGGAGGGCAGCTATTAGG). The protospacers for the target sites were ordered as a pair of oligonucleotides, annealed, and ligated into pX330 using BsmBI (Plk1-CRISPR vector). To knock-in the 3xMyc-tag at the C-terminal coding region of the Plk1 gene, a donor plasmid was created by blunt cloning a gBlock (IDT) containing the 3xMyc-tag in frame with Blasticidin flanked by homology (0.5 kb) of Plk1. To maintain donor integrity, the coding sequence of the targeting region in the donor was made silent (maps for donor plasmids are available upon request). Cells (5 × 10E + 05) were transfected with 1 μg of both, CRISPR vector and donor vector using LTX transfection reagent according to the standard protocol. 96 h later Blasticidin (5 μg/ml) was added. One week later, single cell clones were established by serial dilution and analyzed by PCR using Plk1- and tag-specific primers or western blot.

### Western Blot and antibodies

Cells were transfected with empty Flag, Flag-, Myc- and V5-Plk1 plasmids or siRNA for Bora for 24 h, lysed directly or treated with Noc or Thymidine for 16 h. Cells were released in fresh medium or 10 μM MG132 or 5 μM RO3306 or 1 μM Aurora A inhibitor for the time indicated. Protein extracts of cells were prepared by lysis in RIPA buffer (Sigma) supplemented with protease inhibitors (Complete protease inhibitor cocktail, Roche). Protein extracts (25 μg) were separated by SDS-PAGE and transferred onto PVDF membranes using the TransBlot Turbo Transfer System (BioRad). Blocking of membranes was performed with TBST including 2% BSA. The following antibodies were used at the indicated concentrations: mouse monoclonal Plk1 (F-8:sc-17783) (1:1000), GFP (B-2) (1:1000), Cyclin A (H-432) (1:1000) (all Santa Cruz,

Biotechnology Heidelberg, Germany), β-Actin (AC-15:A1978) (1:200.000), Flag (M2:A8592) (1:1000), (Sigma-Aldrich, Taufkirchen, Germany), PLK1 (35–206, #05–844) (1:1000), Cyclin B1 (#4138) (1:1000), Aurora B (#3094) (1:1000), pS137-PLK1 (#07–1348) (1:1000), phospho-Histone H3 (S10 #06–570) (1:1000) (Millipore, Schwalbach, Germany), Myt (PA5–17665) (1:1000), pMyt-T495 (PA5–12633) (1:1000), GAPDH (ab 9485) (1:1000) (Abcam, Cambridge), V5 (46–0705) (1:1000) (Invitrogen, USA), Calnexin (#610524) (1:1000) (BD, Becton Dickinson, USA), Myc (9B11) (1:1000), Bora (D2B9) (1:1000), pT210-PLK1 (T210) (1:1000), pT288- Aurora A (39D8), (1:1000), Aurora A (D3E4Q) (1:1000), Cyclin E (HE12) (1:1000), (all from Cell Signaling, Frankfurt), HRP-conjugated secondary antibodies (1:5000) (GE Healthcare and Jackson Laboratory), anti-Importin α (# I1784, clone IM-75) (Sigma). The ECL Western Blotting Substrate (Millipore, Germany) was used for detection.

### Size exclusion chromatography

Analytical SEC was performed in phosphate buffer (50 mM Tris pH 7.5, 150 mM NaCl, 20 mM CHAPS, 1 mM DTT) at 4 °C using a Superose 3.2/300 column (GE Healthcare) (injection volume 50 μl; flow rate 50 μl/min; fraction size 50 μl). SEC fractions were quantified by western blotting. Analytical SEC of TDs was performed in KPKCl buffer at 4 °C using a Superdex 10/300 column (GE Healthcare) [32].

### Blue native PAGE

Cells were lysed in 20 μl of ice-cold lysis buffer A (50 mM Tris, pH 8.0, 100 mM NaCl, 0.5 mM TCEP, 2 mM MgCl2, supplemented with 1x Complete and PhosphoSTOP (Roche)). Lysis was performed by mechanical force using a pestle, pipetting and three cycles of freeze and thaw. After the addition of 20 µl lysis buffer B (lysis buffer A containing 40 mM CHAPS) and 1 μl Benzoase (Novagen), samples were incubated for 1 h on ice and subsequently centrifuged for 10 min at 4 °C and 13,000 r.p.m. to remove cell debris. 20 μl of supernatant was supplemented with 10 μl of 3x Native PAGE sample buffer (60% glycerol v/v, 15 mM Coomassie G250) and separated on BN-PAGE Novex 3–12% Bis-Tris protein gel system (Life Technologies) according to the manufacturer’s instructions. The cathode buffer was supplemented with 0.002% Coomassie G250, and the separation was performed at 4 °C for 60 min at 150 V, then 60 min at 250 V.

### Protein crosslinking

In a 20-fold excess approach for the homobifunctional crosslinking using Bis (Sulfosuccinimidyl) suberate (BS3) (20:1 Crosslinker-Protein) we added the appropriate amount of BS3 to lysates. The crosslinking reaction was done for 30 min followed by quenching of unreacted BS3 and by analyzing the products using a polyacrylamide gel and western blotting.

### Subcellular fractionation

All preparations were performed on ice. Cells were washed with PBS, harvested, and resuspended in hypotonic buffer (20 mM Tris-HCl, pH 7.4, 10 mM KCl, 2 mM MgCl_2_, 1 mM EGTA, 0.5 mM DTT, 0.5 mM PMSF and Roche complete protease inhibitors; 300 μl were added per 100-mm tissue culture dish), incubated for 5 min followed by the addition of NP-40 (Nonidet P-40) to a final concentration of 0.1%. Following 3 min of incubation, the cytoplasm and nuclei were separated by centrifuging at 800 g for 8 min. Subsequently, to ensure the removal of nuclear remains, the cytoplasmic fractions were centrifuged at 1500 g for 5 min, and the supernatants were collected as the final cytoplasmic fractions. The nuclei were purified by 10 min incubation in isotonic lysis buffer (20 mM Tris-HCl, pH 7.4, 150 mM KCl, 2 mM MgCl_2_, 1 mM EGTA, 0.3% NP-40, 0.5 mM DTT, 0.5 mM PMSF and Roche complete protease inhibitors and centrifuged at 700 g for 7 min.

### Cell cycle assays

For the cell cycle analysis, cells were harvested, washed with PBS, fixed in chilled 70% ethanol at 4 °C for 30 min, treated with 1 mg/ml RNase A (Sigma-Aldrich), and stained with 100 μg/ml propidium iodine for 30 min. Cell cycle quantification was performed using a FACS Calibur instrument and Cellquest Pro software (both BD Biosciences).

### Confocal microscopy

For indirect immunofluorescence staining, cells were seeded on cover slides. Briefly, cells were treated for the indicated time points, then fixed for 5 min in -20 °C methanol. DNA was stained with DAPI (Roche). Images were taken using an AxioObserver.Z1 microscope with a HCX PL APO CS 63.0 × 1.4 oil UV objective (Zeiss, Göttingen) and a confocal laser-scanning microscope (CLSM, Leica CTR 6500, Heidelberg). The significance of differences between populations of data was assessed according to the Student’s two-tailed test (*p ≤ 0.05; **p ≤ 0.01; ***p ≤ 0.001).

### Immunoprecipitation

Cell lysates were incubated with Protein G Sepharose beads (GE Healthcare) and the specific antibody overnight at 4 °C. Immunoprecipitates were washed 3x with ice-cold buffer (20 mM Tris-HCl [pH 8.2], 150 mM NaCl, 1% [v/v] TritonX-100).

### In vitro kinase assay

This assay using immunoprecipitated PLK1 was performed as described previously [33].

### NanoBiT assay

The NanoBiT: NanoLuc® Binary Technology utilizes an engineered large subunit (LgBiT) and small subunit (SmBiT), 17.6 kDa and 11 aa respectively, to form a functioning luciferase complex. The subunits were fused to N- or C-PLK1. Through protein-protein-interaction (PPI) between the target proteins, the two subunits are brought into close proximity and associate with each other. Live Cell Reagent containing the cell permeable substrate furimazine was added to the sample and led to activation of the luciferase, thus resulting in bioluminescence. HEK 293 T cells were transfected in different combinations of LgBiT and SmBiT vectors. A day later cells were harvested, washed, and resuspended in Opti-MEM®. Subsequently, 2 × 10^4^ cells in 10 μl were transferred onto a 96-well plate. Nano-Glo® Live Cell Substrate was mixed with Nano-Glo® LCS Dilution Buffer (20:1) and applied to the transfected cells. Immediately after application of the Nano-Glo® Live Cell Reagent, luminescence was measured with a luminometer (Victor 1420 Multilabel Counter) indicating PPI between proteins tagged with LgBiT and SmBiT. The significance of differences between populations of data was assessed according to the Student’s two-tailed test (*p ≤ 0.05; **p ≤ 0.01; ***p ≤ 0.001).

### Förster resonance energy transfer (FRET) using Flow Cytometry and Confocal Microscopy

For the FRET measurement via Flow Cytometry HEK 293 T cells were transfected with YFP and CFP constructs. 24 h later cells were trypsinized, once washed with, and resuspended in cold PBS. FRET signals were assayed on a CytoFLEX flow cytometer (Beckman Coulter) equipped with 405 nm, 488 nm, 561 nm, and 638 nm lasers. CFP and FRET cells were excited with the 405 nm laser, emission was taken in the 450/45 BP filter for CFP and 525/40 BP for FRET. For YFP measurement cells were excited by the 488 nm laser and fluorescence was collected with a 525/40 BP filter. CytExpert software (Beckman Coulter) was used for analysis. To apply the “FRET and Co-localization Analyzer”, we cloned PLK1-WT and PLK1-T210E into the mTurquoise 2 vector. For the confocal microscopy, cells were transfected on poly-L-lysine coated coverslips in a 6-well plate for 24 h. The next day cells were fixed for 5 min in −20 °C methanol and mounted on a microscope slide with Vectashield mounting medium. Images were taken with a confocal-scanning microscope (CLSM, Leica CTR 6500 Heidelberg) and analyzed for FRET using “FRET and co-localization analyzer” ImageJ plug-in.

### Immunofluorescence microscopy and antibodies for immunofluorescence

For indirect immunofluorescence staining cells were seeded on cover slides. Briefly, cells were treated for the indicated time points, then fixed for 5 min in −20 °C methanol and permeabilized for 10 min at room temperature with 0.1% Triton X-100. The following primary antibodies were used for staining: PLK1 (F-8) (#sc-17783) (Santa Cruz Biotechnology), PLK1-pT210 (#54725) (Cell Signaling Technology). FITC, and Cy3 conjugated secondary antibodies were obtained from Jackson Immunoresearch (Newmarket, UK). DNA was stained with DAPI (Roche). Images were taken using an AxioObserver.Z1 microscope with a HCX PL APO CS 63.0 × 1.4 oil UV objective (Zeiss, Göttingen) and a confocal laser-scanning microscope (CLSM, Leica CTR 6500, Heidelberg). Images were imported in the software ImageJ Fiji, which was used to measure the localization and the intensities of the different proteins.

### Time lapse microscopy

HeLa mCherry-histoneH2B or PLK1–3xMyc expressing mCherry-histoneH2B were transfected with siRNA against Bora or with the different Bora constructs and synchronized by single or double thymidine in the G1-S boundary. Cells were released for 4 h and then transferred for time-lapse analysis to the microscope stage. Live microscopy was performed with Axioimager inverted Z1 (Zeiss) equipped with an environmental chamber (Zeiss) that maintained the cells at 37 °C in a humidified environment of 5% CO_2_. Images were taken every 10 min using an Axiocam MRm camera (Zeiss) driven by Axiovision SE64 software (Zeiss). Movies and JPEG files were imported into ImageJ and proceeded using the same software. Nuclear envelope breakdown was judged as such when the nuclear membrane lost a smooth and the linear periphery. The first frame showing a pole ward movement of the chromosomes was defined as anaphase onset.

### Statistical methods

All experiments were performed at least in triplicate. Standardization and statistics were determined as described [34]. In brief, statistical analysis was performed using Microsoft Excel and GraphPad Prism software. For paired *t*-tests, all experimental groups were compared with their respective groups. Student**’**s *t*-test was used to determine statistical significance between the two groups. Significant differences (*p ≤ 0.05; **p ≤ 0.01; ***p ≤ 0.001) are indicated in the Figures with asterisks.

## Results

### Dimerization of PLK1 and its subdomains in vitro and in vivo

**Fig. 1:**
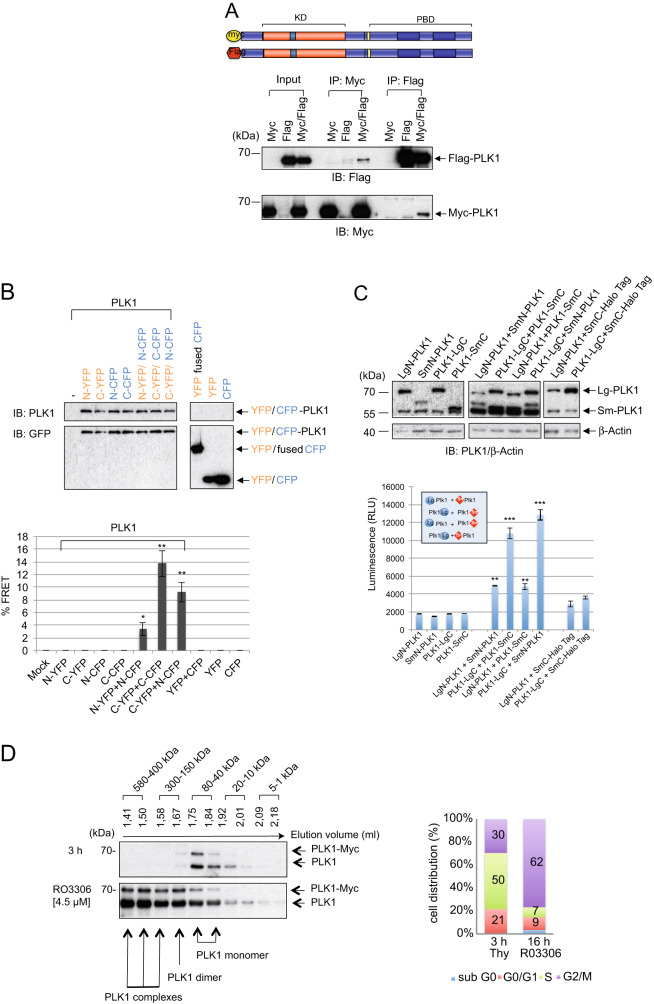
PLK1 oligomerization in vivo and in vitro. **A** (Upper) Schematic representation of the full-length PLK1. (Lower) Lysates of HEK293T cells co-expressing the Myc- and Flag-tagged full-length PLK1 were subjected to IP using Flag- and Myc-specific antibodies followed by western blotting using Flag- or Myc-antibodies. **B** Screening of full-length PLK1 oligomerization by FRET. (Upper) HEK293T cells were co-transfected with N-or C-terminal CFP/YFP-fused PLK1 constructs alone or in combination and 24 h later blotted with anti-PLK1 or anti-GFP antibodies. (Lower) FRET efficiency of three independent experiments was determined. A two-tailed unpaired *t-test* was performed. (*p ≤ 0.05; **p ≤ 0.01). **C** Screening of full-length PLK1 oligomerization by the NanoBiT method. (Upper) Western blot analysis of PLK1 fused to the luciferase subunit LgBiT or SmBiT with anti-PLK1 and anti-β-Actin. (Lower) HEK293T cells transfected with the same combinations used for the western blot experiment were screened for light emission using a luminometer (Victor 1420 Multilabel Counter). A two-tailed unpaired *t-*test was performed. (**p ≤ 0.01; ***p ≤ 0.001). **D** (Left) Size exclusion chromatography of endogenous PLK1. PLK1–3xMyc cells were synchronized to the G1/S boundary using thymidine and released for 3 h or synchronized into the G2-phase using the CDK1 inhibitor RO3306 (4.5 µM). The protein extracts were loaded onto a Superose 3.2/300 size exclusion column and fractionated at a flow rate of 50 µl/min. The factions were resolved by SDS-PAGE and probed with anti-PLK1. (Right) The cell cycle distribution of synchronized HeLa cells is represented as a bar graph.

To investigate the regulation of PLK1 in human cells, we first generated differently tagged full-length, wild-type (WT) constructs of PLK1 (Flag-PLK1 and Myc-PLK1) and tested their ability to oligomerize using co-immunoprecipitation (co-IP) experiments. Upon performing a co-transfection in 293 T cells, the co-IP using Myc- or Flag-specific antibodies resulted in the precipitation of the corresponding PLK1 variant indicating the ability of full-length PLK1 to oligomerize in cells (Fig. 1A). The specificity of PLK1 oligomers was supported by control IPs involving differently tagged PLK1 (Fig. S1A) or PLK1 and unrelated proteins such as SKAP, LAT, or RIAM (Fig. S1B). PLK1 is associated specifically with itself or with SKAP, but not with RIAM and LAT as reported previously [35]. To identify the PLK1 domain involved in the oligomerization, we similarly generated differently tagged versions of the kinase domain (Myc- and V5-PLK1 KD) and the PBD (Flag-and Myc-PLK1 PBD), respectively. Subsequently, their ability to oligomerize was tested in co-IPs. Interestingly, both KD and PBD were able to oligomerize when co-transfected in 239 T cells (Fig. S1C, D).

Next, we tested our model of PLK1 oligomerization in living HeLa cells by FACS-based fluorescence energy resonance transfer (FRET) assays. We fused CFP and YFP onto either the N-terminal (N-YFP- or N-CFP-PLK1) or the C-terminal domain (C-YFP- or C-CFP-PLK1) of PLK1. Following co-expression, we measured the FRET emission in cells by FACS (Fig. 1B and S1E). While FRET signals were not detected in control cells (CFP, YFP, or CFP/YFP), we found a 10–14-fold increase in FRET signals in cells expressing C-CFP-PLK1 and C-YFP- PLK1 or C-YFP-PLK1 and N-CFP-PLK1, supporting the oligomerization of PLK1 in living cells. Next, we applied the NanoBit method [36] and expressed one or two PLK1 variants fused at the N- or C-terminal ends to LgBiT or SmBit. The luminescence originating from protein-protein interactions that reconstitute the complete luciferase complex was determined (Fig. 1C). The expression of two C-tagged PLK1 variants yielded strong signals supporting PLK1 oligomerization. Interestingly, both assays, FRET and NanoBit, showed that N-terminal tagging with CFP/YFP or LgBiT attenuated FRET emission compared to C-tagged PLK1 suggesting that the N-terminal region, including the KD, plays a crucial role for oligomerization (Fig. 1B, C).

Further, to investigate whether cellular PLK1 also oligomerizes, we exploited the CRISPR/Cas9 method to generate a HeLa cell line in which a 3xMyc-tag was added to endogenous PLK1 (Fig. S2A). We selected clones harboring one tagged *PLK1* allele, from here on dubbed PLK1–3xMyc cells. Thus, in the case of PLK1 oligomers, we were able to discriminate in Myc-IPs two forms of PLK1 that differ in size. However, as HeLa cells contain three copies of chromosome 16, where PLK1 is localized [37], our knock-in experiments could probably target only one allele, leaving the remaining two PLK1 alleles unmarked. The PLK1–3xMyc cells were synchronized to the G1/S boundary using thymidine and released for 3 h or synchronized into the G2-phase using the CDK1 inhibitor RO3306 [38]. Afterward, the protein lysates of synchronized cells were subjected to size exclusion chromatography (SEC) (Fig. 1D). The elution profiles were analyzed by Western blotting and compared with those of protein standards (Table 1). Most of the PLK1 protein appeared in the S phase predominantly in the fraction with an elution volume of 1.75–1.84 ml. According to the elution profile of protein molecular mass standard (Table 1), these fractions contain protein ranging in size between ~80–40 kDa (Fig. 1D upper panel), indicating that during the S-phase PLK1 with a molecular weight of 66 kDa is present as a monomer in cells. The SEC profile of lysates derived from RO3306-synchronized cells that are exclusively in G2 showed PLK1 to be present in the elution fraction of 1.67 ml (of ~150 kDa), proposing that PLK1 seems to exist as a dimer in the G2 phase (Fig. 1D, bottom panel). Furthermore, PLK1 also appeared in other fractions suggesting the presence of PLK1 in complexes of higher molecular weight and small amounts of monomeric PLK1, respectively (Fig. 1D, bottom panel). The SEC results indicate that during the G2 phase, endogenous PLK1 might adopt a homodimeric conformation prompting us to evaluate the model of dimeric PLK1 in more detail.

**Table 1:** SEC-based separation of molecular weight standard.

Calibration		
Protein	Elution volume (ml)	Molecular weight Mr (Da)
Thyroglobulin	1.349	669000
Ferritin	1.509	440000
Aldolase	1.66	158000
Convalbumin	1.743	75000
Ovalbumin	1.791	43000
Carbonic Anhydrase	1.871	29000
Ribonuclease A	1.959	13700
Aprotinin	2.039	6500

### Bora sustains PLK1 dimerization during the G2 phase

**Fig. 2:**
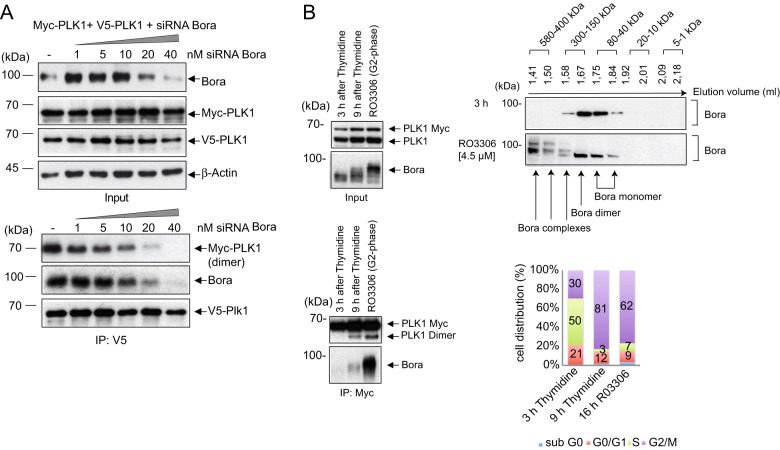
Bora promotes PLK1 dimerization in vitro and in vivo. **A** Bora promotes PLK1 dimerization. (Upper) HEK293T cells were transfected with increasing amounts of Bora siRNA and 24 h later transfected with Myc- and V5-PLK1. (Lower) Lysates were subjected to co-IP using V5- antibodies and western blotting using Bora-, V5, Myc- or ß-Actin-antibodies. **B** Size exclusion chromatography (SEC) of endogenous Bora. PLK1–3xMyc cells were synchronized to the G1/S boundary using thymidine and released for 3 h or 9 h or synchronized into the G2-phase using the CDK1 inhibitor RO3306. (Upper left panels) Lysates of the synchronized cells were subjected to an SDS-PAGE and stained with anti-PLK1, and anti-Bora antibodies. (Lower left panels) The lysates were subjected to IP using Myc-specific antibodies followed by western blotting with anti-Bora or PLK1 antibodies. (Upper right panels) The protein extracts of synchronized PLK1–3xMyc cells were loaded onto a Superose 3.2/300 size exclusion column and fractionated at a flow rate of 50 µl/min. The factions were resolved using an SDS-PAGE and probed with anti-Bora antibodies. (Lower right panel) The cell cycle distribution of synchronized HeLa cells is represented as a bar graph. (*n* = 3).

Mass spectrometry (MS) analysis performed by Seki et al [25]. discovered that PLK1 is a major Bora-interacting protein during the G2 phase and that the Bora-PLK1 complex is already detectable at the S-G2 transition, which is timely ahead of PLK1-T210 phosphorylation and kinase activation that peak during late G2-M. In light of these findings and of Bora’s functional role in the regulation of PLK1 activity, we sought to investigate whether Bora might support the oligomerization of PLK1 during early G2. First, we co-expressed Myc- and V5-PLK1 in Bora-depleted cells. In the absence of Bora, we observed a substantial decrease in self-associated Myc-PLK1 in V5-PLK1-IPs (Fig. 2A, lower panel). To deepen our understanding of Bora’s role in PLK1 oligomerization, we further incubated cell lysates co-expressing PLK1 KD- or PLK1 PBD-tagged constructs with increasing amounts of purified GST-Bora. Co-IP experiments showed that increasing amounts of Bora did not improve the KD oligomerization (Fig. S2B) but enhanced, even at low concentrations, by almost 60% the oligomerization of the PBD (Fig. S2C, lane 1 vs. 2). It is likely that even at low concentrations, GST-Bora already achieved a saturating effect for PBD oligomerization, which may explain the little enhancement in oligomerization induced by higher Bora concentrations suggesting a scaffold function of Bora for PLK1 oligomerization (Fig. S2C, lanes 3–5). Furthermore, we incubated the lysate of HeLa cells expressing differently tagged-PBD domains with a His-Bora fusion protein and performed a Myc-PBD-IP (Fig. S2D). With increasing concentrations of His-Bora, we could raise the amount of PLK-PBD oligomerization, confirming Bora’s role in inducing PLK1 PBD oligomerization. Finally, in cells co-expressing full-length Myc-PLK1 and V5- PLK1, increasing GST-Bora concentrations resulted in rising oligomerization of the PLK1 as indicated by the amount of precipitated V5-PLK1 (Fig. S2E, left panel). By contrast, the incubation of differently tagged-PLK1 with GST alone did not change the PLK1 oligomerization status (Fig. S2E, right panel*)*. These results corroborate that Bora stimulates the oligomerization of PLK1 in cells, likely, by supporting the oligomerization of the PBD domains of two PLK1 monomers.

To deepen our knowledge of Bora’s putative role for PLK1 oligomerization, we co-expressed in 293 T cells differently tagged full-length Bora (Flag-Bora and Myc-Bora) and performed a Myc- or Flag-IP (Fig. S2F). We found the corresponding Bora variant in both co-IPs indicating Bora’s oligomerization ability. The kinetics of Bora association in synchronized cells released from a G1-S arrest was monitored by Native PAGE of cellular lysates. Considering posttranslational modifications of Bora, this assay indicates that the Bora protein (MW ~80 kDa) migrates as monomeric form, as dimeric form (146–160 kDa), and as Bora complexes of higher molecular weight (Fig. S2G). SEC experiments confirmed the presence of Bora dimers in the fraction with an elution volume of 1.67 ml (~ 150 kDa) during the S- and G2-phase (Fig. 2B, right upper panel). However, Bora was present as higher molecular weight complexes (~ 300 kDa) exclusively in the G2-synchronized lysates. Interestingly, both proteins, PLK1 and Bora, appeared in this fraction, suggesting the presence of PLK1 dimers and Bora dimers at the same time in cells (Fig. 1D, Fig. 2B). This data provides evidence that dimeric Bora co-exists with PLK1 dimers during the G2 phase.

Our previous experiments indicate Bora’s role in supporting PLK1 oligomerization, and since Bora is a cytoplasmic protein exclusively, we expected that the cytoplasmic pool of PLK1 undergoes a Bora-dependent dimerization during the G2-phase. Thus, different lines of evidence derived from independent experimental approaches (FRET, NanoBit, SEC, Native PAGE, Bora IP) suggest that during the G2 phase, cytoplasmic PLK1 exists as a dimer in an association with Bora. Thus, we will refer from now on to the oligomerized form of PLK1 (~120–140 kDa) as dimer.

### Phosphorylation of PLK1-T210 inhibits the Bora-induced dimerization of PLK1

**Fig. 3:**
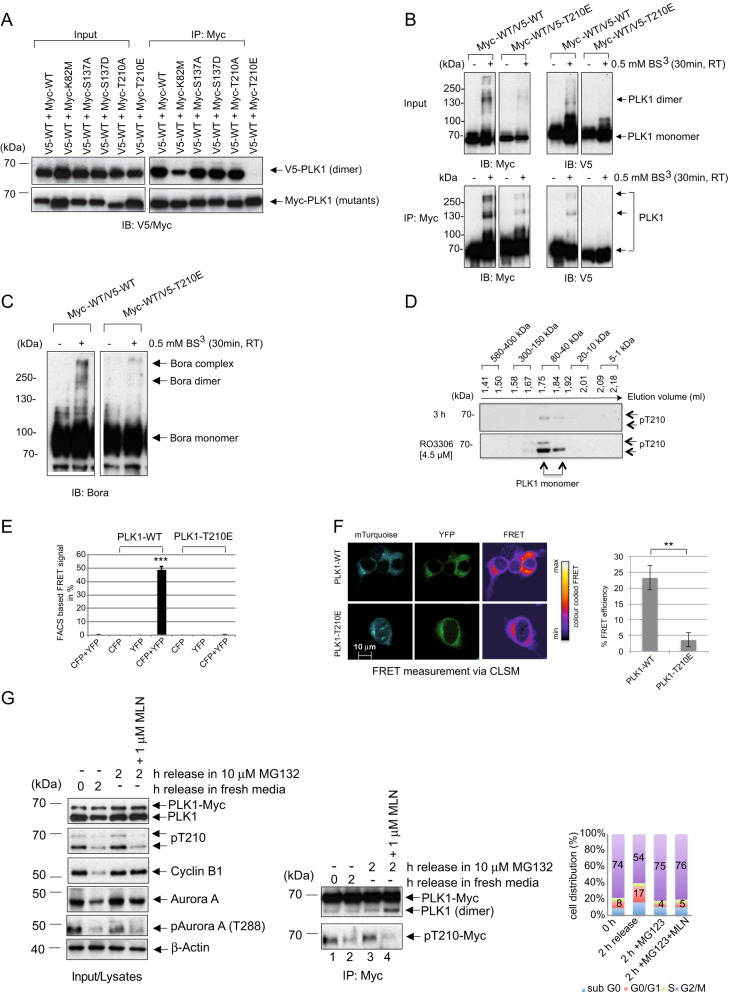
Phosphorylation of PLK1-T210 blocks PLK1 dimerization. **A** The T210 phospho-mimicking mutation blocks PLK1 dimerization. Lysates of HEK293T cells co-expressing V5-tagged full-length PLK1 (wild-type) and Myc-tagged full-length PLK1 mutants (K82M, S137A/D, T210A/E) were subjected to IP using a Myc-specific antibody followed by western blotting with V5-and Myc-antibodies. **B**, **C** Bis(Sulfosuccinimidyl) suberate (BS3) (20:1 Crosslinker-Protein) was added to lysates of HEK293T cells expressing Myc/V5- tagged-PLK1-WT and PLK-T210E. The crosslinking was performed for 30 min followed by quenching of unreacted BS3. Afterward, IPs using anti-V5 and anti-Myc antibodies were carried out. The products of the crosslinking were analyzed by SDS-PAGE and western blotting with PLK1- and Bora-specific antibodies. **D** Size exclusion chromatography of endogenous PLK1. PLK1–3xMyc cells were synchronized to the G1/S boundary using thymidine and released for 3 h or synchronized into the G2-phase using the CDK1 inhibitor RO3306. The protein extracts were loaded onto a Superose 3.2/300 size exclusion column and fractionated at a flow rate of 50 µl/min. The factions were resolved using an SDS-PAGE and probed with the anti-PLK1-pT210 antibody. **E** HEK293T cells were co-transfected with C-terminal CFP/YFP-fused PLK1 and -PLK1-T210E constructs alone or in combination. The FRET efficiency for the indicated constructs and combinations from three independent experiments was determined. A two-tailed unpaired t-test was performed (***p ≤ 0.001). **F** (Left) Confocal images of HEK293T. Cells grown on coverslips, co-transfected with YFP and mTurquoise PLK1-WT or PLK1-T210E were analyzed for FRET using the FRET co-localization analyzer ImageJ plugin. FRET-images give the calculated amount of FRET for each pixel in the merged images. The ImageJ plugin color codes the relative FRET efficiency, which is indicated by the displayed color bar. Scale bars: 10 μm. (right) Quantifications of mTurquoise/YFP emission ratios of cells expressing PLK1-WT or PLK1-T210E. Error bars indicate the standard deviation based on the emission of 10 individual cells. A two-tailed unpaired *t*-test was performed. (**p ≤ 0.01). **G** The phosphorylation of T210 by Aur-A abolishes PLK1 dimerization. (Left) Mitotic HeLa cells expressing PLK1–3xMyc enriched by Nocodazole (Noc) treatment and a shake-off were released in 10 μM MG132 or 10 μM MG132/1 μM MLN for 2 h. Lysates were resolved by SDS‐PAGE and immunoblotted for PLK1, PLK1-pT210, Cyclin B1, Aur‐A, and Aurora-pT288. (Middle) Lysates of HeLa-PLK1–3xMyc cells were subjected to IP using anti-Myc and immunoblotted for PLK1 and PLK1-pT210. (Right) The cell cycle distribution of synchronized HeLa cells represented as a bar graph.

Previous FRET and NanoBit assays (Fig. 1B, C) indicated a critical role of the PLK1 KD in PLK1 dimerization. To shed light on this aspect, we performed site-directed mutagenesis of critical functional residues (K82, S137, T210) within the KD of Myc-tagged PLK1 full-length for co-transfection with V5-PLK1 WT (Fig. S3A). The co-IPs showed that all PLK1 mutants could dimerize, except for the kinase-inactive PLK1-K82M that reduced the dimerization. Most intriguingly, the phosphomimetic mutant PLK1-T210E almost totally abolished the dimerization of PLK1 (Fig. 3A). Using insect cells (SF9) expressing PLK1-WT and PLK1-T210E, we could confirm, using co-IPs, the inability of PLK1-T210E to dimerize (Fig. S3B).

To investigate the role of PLK1-T210 in the Bora-stimulated PLK1 dimerization, we performed BS3-mediated crosslinking experiments in lysates and co-IPs of cells expressing Myc/V5- tagged-PLK1-WT and PLK-T210E (Fig. 3B). Crosslinked-PLK1 was observed only when wild-type PLK1-WT (Myc/V5-tagged) was expressed, but not when PLK1-T210E was co-expressed with PLK1-WT, indicating that mimicking T-loop phosphorylation disrupts PLK1 dimerization (Fig. 3B). In Myc/V5-IPs followed by Western blot experiments, we observed a band of approx. 140 kDa indicating PLK1 as a dimer (Fig. 3B, lower panel). Interestingly, we also noticed additional bands representing PLK1 in higher molecular weight complexes (> 250 kDa). Furthermore, we probed the same PLK1 crosslinking experiment with Bora antibodies. We observed Bora’s presence in the same PLK1-WT crosslinking complex > 250 kDa (Fig. 3C) but not when the crosslinking experiments were performed with the PLK1-T210E mutant (Fig. 3C), supporting the idea that mimicking T210 phosphorylation might disrupt the Bora-supported PLK1 dimerization. In line with these observations, probing the size exclusion chromatography (SEC) fractions with PLK1-pT210 specific antibodies showed a strong phospho-signal in the RO3306-treated cell lysate compared with the S-phase lysate (Fig. 3D). Most importantly, the pT210 signal was only present in the fraction with an elution volume of 1.75–1.84 ml (~80–40 kDa), where monomeric PLK1 is detectable, corresponding with a molecular weight of ~80–40 kDa. The PLK1-pT210 signal is absent in the fraction containing PLK1 dimer or PLK1 dimer complexed to Bora dimer (elution volumes 1.67–1.58 ml corresponding to molecular weights of ~150–300 kDa) (Fig. 1D, Figs. 2B, 3D). These results strengthen the model that mimicking PLK1-T210 phosphorylation abrogates PLK1 dimerization during the G2 phase.

For further validation, we performed a FRET assay using C-tagged (CFP, YFP) forms of PLK1. While PLK1 expression resulted in a high FRET emission, PLK1-T210E hampered the signal intensity in cells (Fig. 3E, S3C). Similarly, microscopy-based FRET indicated that the emission intensity in cells expressing PLK1-T210E decreased by 6–8 folds compared to cells expressing WT PLK1 (Fig. 3F, S3D). All this supports the critical role of the PLK1 KD and confirms that mimicking phosphorylation of T210 impedes PLK1 dimerization. As the phosphorylation of PLK1-T210 during G2-M is implemented by Aur-A [25, 27, 39], we were interested in investigating the role of Aur-A in PLK1 dimerization. Nocodazole-synchronized PLK1–3xMyc cells were released for 2 h in the presence of MG132 with or without the Aur-A inhibitor MLN8237 [40]. Mitotic cells treated with MLN8237 showed a reduced Aur-A autophosphorylation (Aur-A-pT288) and decreased PLK1-pT210 (Fig. 3G, left panel). Interestingly, the loss of PLK1-pT210, inherent to Aur-A inhibition, matched with an increase in PLK1 dimerization in the Myc-IP (Fig. 3G, right panel, lane 4). Further, we incubated in a cold kinase assay Aur-A kinase with Bora and PLK1-WT. In the absence of Aur-A kinase, PLK1-WT lacking T210 phosphorylation could dimerize (Fig. S3E, right panel). After the incubation with Aur-A kinase, PLK1 could readily be phosphorylated at T210, concomitant with a PLK1 dimer dissociation (Fig. S3E, right panel). Taken together, the data suggest that the Aur-A phosphorylation of PLK1-T210 during late G2 represents a crucial step fostering the dissociation of PLK1 dimer and shifting PLK1 towards the active monomer with phosphorylated T210.

The previous experimental results hinted at a difference in the catalytic activity between dimeric and monomeric PLK1. To study this, we investigated the global PLK1 activity directly in living cells using a PLK1-specific FRET-based biosensor [27] (Fig. S3F, left panel). In 293 T cells, phosphorylation of the biosensor by PLK1 caused a change in the CFP/YFP emission ratio (Fig. S3F, upper right panel). This assay using living cells expressing PLK1 WT or PLK1- T210E confirmed that cells expressing PLK1-T210E with strong PLK1 activity are void of dimers compared to cells with low PLK1 activity exhibiting high levels of dimeric PLK1 (Fig. S3F, lower panel).

### PLK1 dimerization in time and space

**Fig. 4:**
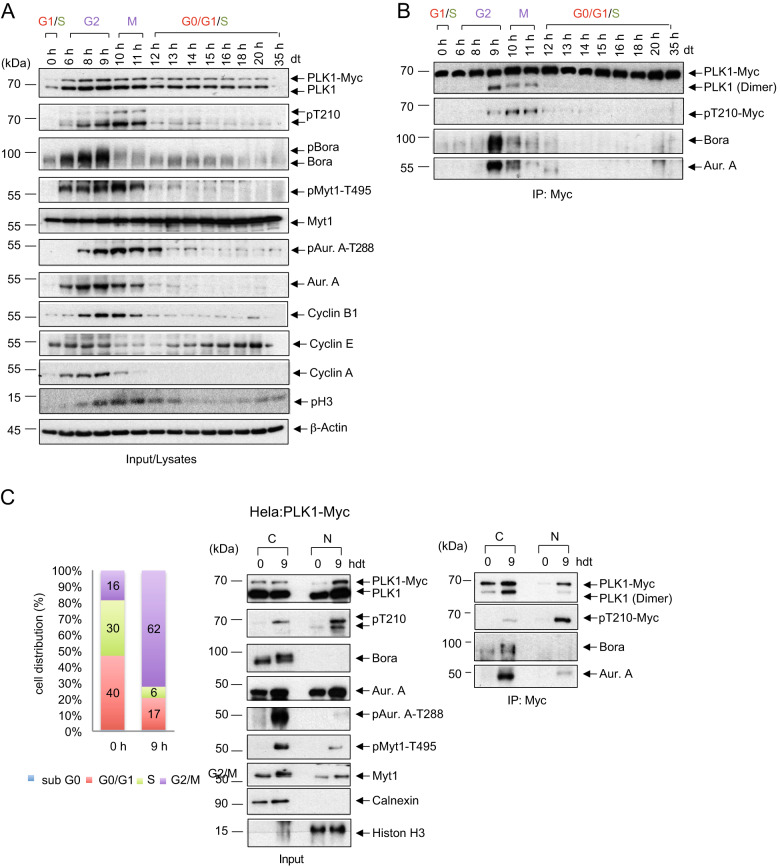
Dimerization of PLK1 in space and time during the cell cycle. **A** HeLa-PLK1–3xMyc cells were synchronized at the G1/S boundary by a double thymidine arrest (dt), released into fresh medium, and harvested at the indicated times. The accumulation of key protein markers was used to determine the cell-cycle stages. The levels of the indicated proteins were analyzed by western blotting. **B** IPs using anti-Myc antibody from HeLa-PLK1–3xMyc cell lysates were blotted for PLK1, PLK1-pT210, Bora, and Aur-A. **C** HeLa-PLK1–3xMyc cells were synchronized at the G1/S boundary by dt treatment and released for 9 h. (Left) FACS analysis of the cell populations. (Middle) Cell lysates were fractionated, subjected to immunoblotting for PLK1, PLK1-pT210, Bora, Aur-A, pAur-A T288, pMyt1-T495, Myt1, Calnexin, and Histone H3. (Right) Fractionated lysates were subjected to anti-Myc co-IP and blotted for PLK1, PLK1-pT210, Bora, and Aur-A*.*(*n* = 3).

We determined the kinetics of PLK1 dimerization in synchronized PLK1–3xMyc cells released from a G1-S arrest (Fig. 4A). The expression and phosphorylation profiles of critical cell cycle regulators (PLK1, Cyclin B1, pH3) were similar to WT cells, indicating that the 3xMyc-tail of endogenous PLK1 did not interfere with normal cell cycle progression (Fig. 4A, S4A, B). We observed a gradual accumulation of Bora levels starting from the S phase (6 h) with a peak at G2 (9 h) followed by a steep decrease once PLK1 becomes fully activated (PLK1-pT210 at 10 h), which is in agreement with the role of PLK1 in promoting the proteasomal degradation of Bora during early mitosis (Fig. 4A) [41]. The corresponding Myc-co-IP revealed peak levels of dimeric PLK1 during the G2 phase. PLK1 dimer and the T210 phosphorylation exhibited an inverse correlation (Fig. 4B). Remarkably, the co-IP at 9 h (G2 phase) indicated that maximal levels of PLK1 dimers, along with very low levels of pT210, were found in a ternary complex with high levels of Bora and activated Aur-A (pT288) (Fig. 4A, B), implying that PLK1 dimerization prevents the early availability of T210 for Aur-A phosphorylation. To investigate this aspect in more detail, we performed a Myc-Co-IP in the nucleus- and cytosol- fractionated PLK1–3x-Myc cell lysates that were either synchronized to the G1-S boundary (0 h) or released in the G2 phase (9 h after thymidine release) (Fig. 4C, left and middle panels). The Myc-co-IP confirmed the presence of dimeric PLK1 exclusively in the cytoplasm in the G2 phase, while the nucleus contains the monomeric and active form of PLK1 (pT210) (Fig. 4C, right panel). However, in contrast to the nuclear, active, and monomeric PLK1, the cytoplasmic and dimeric PLK1, despite its association with active Aur-A (pT288) and phosphorylated Bora (as indicated by the slow migrating band), exhibited only residual pT210 signal, suggesting that the dimerization of PLK1 seems to contribute to the timely activation of the cytoplasmic PLK1 during G2 phase (Fig. 4C, middle and right panels).

### Enforcing PLK1 dimer impedes the G2-M transition by hampering the PLK1-Bora-Aur-A axis

**Fig. 5:**
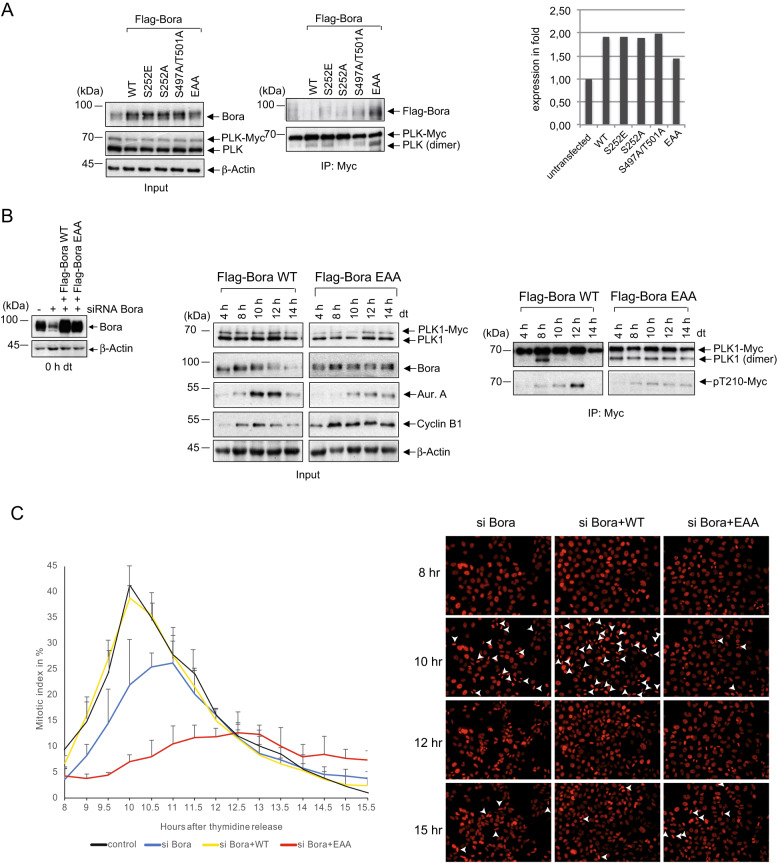
Blocking the timely defined dissociation of PLK1 dimers impedes the G2-M transition. **A** (Left) HeLa-PLK1–3xMyc cells were transfected with different Flag-tagged Bora variants, Bora WT, Bora S252E, Bora S252A, Bora S497A/T501A (Bora AA), and the triple mutant Bora S252E/S497A/T501A (Bora EAA). Cells were synchronized by thymidine treatment at the G1-S boundary and released for 8 h to reach the G2 phase. Cell lysates were subjected to immunoblotting for Bora, PLK1, and ß-Actin. (Right) The relative amounts of exogenously expressed Bora variants were quantified. (Middle) Lysates of transfected HeLa-PLK1–3xMyc cells were subjected to IP using anti-Myc and immunoblotted as indicated. **B** (Left) Bora-depleted HeLa-PLK1–3xMyc cells were rescued with Flag-tagged Bora WT or Bora EAA and synchronized by thymidine arrest to the G1-S boundary. Cell lysates were immunoblotted for Bora and β-Actin. (Middle) HeLa-PLK1–3xMyc rescued with Flag-tagged Bora WT or Bora EAA were synchronized by thymidine treatment at the G1-S boundary and released for the indicated time points. Cell lysates were immunoblotted for PLK1, Bora, Aur-A, Cyclin B1, and β-Actin. (Right) The lysates were subjected to anti-Myc IP and blotted for PLK1 and PLK1-pT210. **C** Bora-depleted HeLa-PLK1–3xMyc cells expressing mcherry-Histone H2B cells were rescued with Flag-tagged Bora WT or Bora EAA, synchronized by thymidine treatment at G1/S, and released for 16 h. (Left) The mitotic indices have been assessed using time-lapse microscopy. The percentages of mitotic cells over time in each treatment group is represented in the line diagram. The results are presented as means ± SD (*n* = 150). (Right) Representative Figures of time-lapse analysis. The arrowheads point to mitotic cells.

Previously, we demonstrated that Bora promotes PLK1 dimerization. Furthermore, treatment of lysates of G2 synchronized PLK1–3xMyc cells with γ-phosphatase reduced the dimerization of PLK1 along with dissociation of the Bora-PLK1 complex in the Myc-Co-IP (Fig. S5A), suggesting that PLK1 dimerization during the G2 phase could require phosphorylated Bora protein. Additional analyses showed that the CDK1-mediated priming of Bora might be important for PLK1 dimer assembly as the treatment of G2-synchronized PLK1–3xMyc cells with the CDK1 inhibitor RO-3306 abolishes the association PLK1-Bora and decreases PLK1 dimers in the Myc-IP (Fig. S5B). Unlike the unphosphorylated Bora that weakly associates with the PBD, pre-phosphorylation of Bora-S252 by CDK1 strongly enhances the Bora-PLK1 interaction [42]. To investigate the putative role of Bora S252 in PLK1 dimerization, we performed replacement experiments with Bora mutants (S252A, S252E). Only the Bora S252E mutant stabilized PLK1 dimerization in IP experiments suggesting a role of S252 phosphorylation in PLK1 dimerization (Fig. S5C). To investigate the cellular role of the conformational switch from dimer to monomer during the G2-M transition, we sought to artificially establish a constitutive PLK1 dimer using the Bora mutant that stabilizes the PLK1 dimer (Bora S252E) in combination with a degradation-resistant mutant of Bora (S497A/T501A) (Bora AA). We next expressed the different Flag-Bora mutants in PLK1–3xMyc cells, followed by synchronization in the G2 phase (8 h) (Fig. 5A). Transfected cells expressing exogenous Bora mutants had a ratio of 1–2:1 to endogenous Bora. According to a previous report, low levels of exogenous Bora AA show similar G1, S, and G2 kinetics compared to wild-type cells and still allow PLK1 or Aur-A to ensure their subcellular localization [41]. Myc-IP in these G2-lysates showed that expression of Bora S252E/T497A/T501A (Bora EAA) led to the highest level of PLK1 dimer (Fig. 5A, right panel).

To assess the consequences of a constitutive PLK1 dimer on the G2-M transition, we stabilized PLK1 dimers by rescuing Bora-depleted PLK1–3xMyc cells with physiological levels of Flag-tagged Bora WT and EAA (Fig. 5B, left panel). We determined the kinetics of PLK1 dimer accumulation in rescued and synchronously released cells from a cell cycle arrest at G1-S (Fig. 5B, middle panel). Exogenous Bora WT showed normal degradation kinetics during early mitosis (10 h) and did not affect mitotic entry or exit as indicated by the normal accumulation and degradation of Cyclin B1. The corresponding Myc-IP suggested the presence of maximal PLK1 dimer during G2 (8 h) that dissociate with increasing PLK1-pT210 signal confirming previous results (Fig. 5B, right panel). The rescue of Bora-depleted cells with the degradation-resistant mutant Bora EAA led to a constitutive PLK1 dimerization seen during the entire observation period (Fig. 5B, right panel). More intriguingly, stable PLK1 dimers only showed residual pT210 signal over the 14 h release, and this stabilization did not interfere with Cyclin B1 accumulation during G2. However, Cyclin B1 expression kept steady along with the whole kinetics (Fig. 5B, middle panel). This suggests that either the cells face problems to enter or exit mitosis. To clarify this, we performed time-lapse microscopy. We performed the analysis in synchronized HeLa-PLK1–3x-Myc cells rescued with Bora mutants and expressing mCherry-Histone H2B. Bora depletion predominantly delayed mitotic entry (Fig. 5C) in line with a previous study [25]. The rescue with Flag-Bora-WT restored similar kinetics as in control cells (Fig. 5C). Intriguingly, stabilizing PLK1 dimer (Bora EAA) drastically reduced the mitotic index, hinders the cells from overcoming the G2 phase (Fig. 5C).

PLK1 is not indispensable for normal mitotic entry but instead becomes essential in recovering from DNA damage [2, 43]. Hence, we wondered how stabilizing PLK1 dimers interfered with the transition G2-M and asked whether, through enforcing PLK1 dimers, we altered the dynamics of the entire complex PLK1-Bora-Aur-A, and thus impaired mitotic entry.

**Fig. 6:**
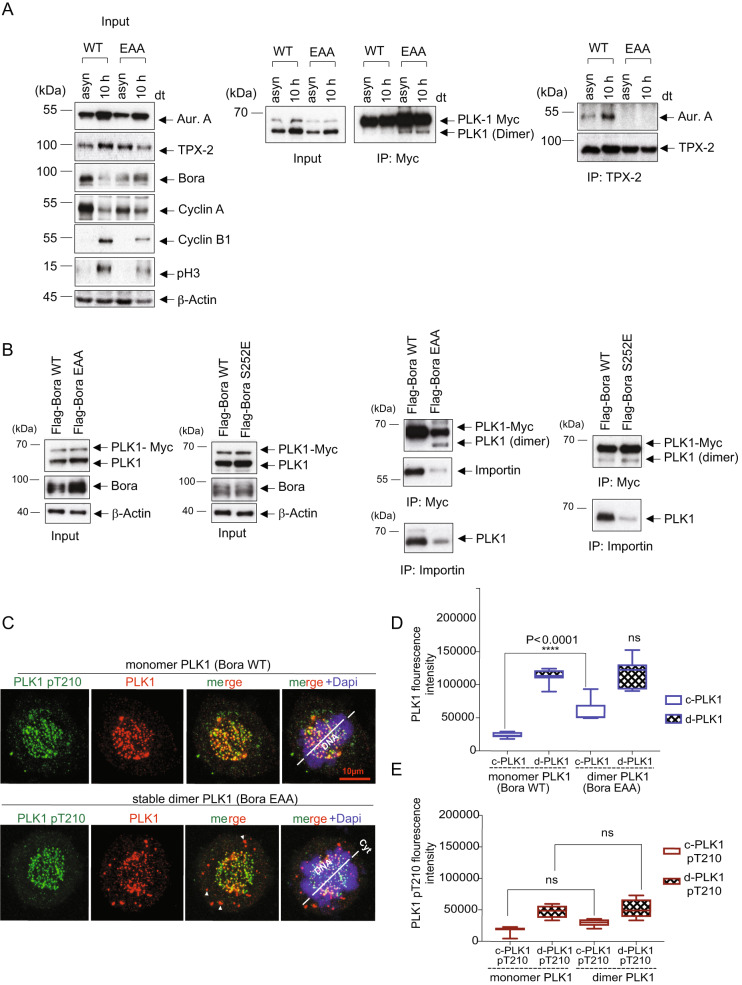
Preventing PLK1 dimer dissociation interferes with PLK1 nuclear transport during the G2 phase and causes PLK1 mislocalization. **A** Stabilizing PLK1 dimers impedes Aur-A-TPX2 interaction. HeLa-PLK1–3xMyc cells were first transfected with Flag-Bora WT or Flag-Bora EAA. Cells were synchronized by thymidine treatment at the G1-S boundary and released for 10 h to reach mitosis. As a control experiment, similarly transfected but asynchronized cells were used. (Left) The cell lysates were first immunoblotted for the indicated antibodies. (Middle) The lysates were subjected to an anti-Myc IP and blotted for PLK1. (Right) The same lysates underwent an anti-TPX2 co- IP, and the precipitates were stained for TPX2 and Aur-A. (*n* = 3). **B** HeLa-PLK1–3xMyc cells were transfected with different Flag-tagged Bora variants, Bora WT, Bora S252E or Bora EAA, followed by a synchronization into the G2 phase using RO3306 (4.5 µM). (Left) Cell lysates were subjected to immunoblotting for Bora, PLK1, and ß-Actin. (Right) Lysates of transfected HeLa-PLK1–3xMyc cells were subjected to IP using anti-Myc or anti-Importin α and immunoblotted for Importin α and PLK1. (*n* = 3). **C** Confocal images of HeLa-PLK1–3xMyc. The cells were first transfected with Flag-Bora WT or Flag-Bora EAA. Cells were synchronized by thymidine treatment at the G1/S boundary and released for 10 h to reach mitosis. Next, the cells were fixed and processed for IF using PLK1 and PLK1-pT210 antibodies. The cytoplasmic and DNA-associated PLK1 and the PLK-pT210 signals are shown as representative Figures. The arrowheads show displaced and secluded PLK1 in the cytoplasm. Scale bar: 10 µm (*n* = 25). **D**, **E** The cytoplasmic and DNA-associated PLK1 and PLK1-pT210 intensities in both transfection groups were quantified. The values were calculated from at least 30 cells and represented as a Box-Whiskers plot (****p ≤ 0.001).

Aur-A and its co-factor TPX2 are key players in the chromatin-driven spindle assembly during early mitosis [44, 45]. Once cells enter mitosis, the PLK1-Bora-Aur-A complex dissociates in a PLK1 phosphorylation-dependent manner [46, 47]. This event sets Aur-A free to interact with its spindle-associated and co-activator TPX2 that re-localizes Aur-A to mitotic spindle [47, 48]. However, by preventing PLK1 dimer dissociation, Aur-A remains trapped with PLK1 and Bora, which eventually prevents it from being recruited to other cellular localization and ultimately hinders mitotic onset. To test this, PLK1–3xMyc cells stably expressing Bora WT and EAA were released from G1-S arrest for 10 h to reach mitosis (Fig. 6A, left panel). Myc-IP confirmed that expression of low levels of Bora EAA stabilized PLK1 dimer within mitosis (Fig. 6A, middle panel). Remarkably, TPX2 co-IPs in mitotic lysates showed that while the active PLK1 monomer (Bora WT) displayed a strong TPX2-Aur-A association after 10 h, enforced PLK1 dimers (Bora EAA) strongly impeded mitotic TPX2-Aur-A interaction, which implies a reduced association of Aur-A with the mitotic spindle (Fig. 6A, right panel).

Based on our data, we suggest that an untimely existing PLK1 dimer maintains the kinase at low activity and prevents PLK1 activity in the cytoplasm to reach the threshold required during the late G2 to support the mitotic transition. Furthermore, blocking the dissociation of PLK1 dimers at the late G2 phase caused cytoplasmic sequestration of Aur-A as indicated by the reduced TPX2-Aur-A association. Consequently, the G2 arrest observed in Fig. 5B, C might only partially be ascribed to blocking the dimer-monomer switch of PLK1 and is more likely to be caused by the dysregulation of the entire PLK1-Bora-Aur-A regulatory axis.

### A constitutive PLK1 dimer restrains the Nuclear Localization Signals (NLS) accessibility to importins, causing PLK1 mislocalization

Previous studies reported that the nuclear translocation of PLK1 during interphase is ensured through a bipartite-NLS located at the kinase domain and by a second NLS localized in the PLK1 PBD sequence [49–51]. According to these studies, the proper nuclear localization of PLK1 during interphase is essential for the initiation and mitosis progression, consequently disrupting both PLK1 NLS arrests cells in the G2 phase. Our experiments showed that the dimerization of full-length PLK1 is based on the kinase domain dimerization and the Bora-induced PBD oligomerization. This prompted us to ask whether PLK1 dimerization during the G2 phase modulates PLK1’s NLS function, thereby regulating the accessibility to the importing complex. To test this, we synchronized the HeLa- PLK1–3xMyc cells expressing low levels of Bora EAA or Bora S252E to stabilize the PLK1 dimer or Bora WT into the G2 phase using RO-3306. Remarkably, the Myc-PLK1-IP and the reverse Importin-IP revealed that importin associates exclusively with monomeric PLK1 (Bora-WT) and is utterly absent in the stable dimer PLK1 co-IP (Bora-EAA and Bora S252E) (Fig. 6B). This result suggests that PLK1 dimerization contributes to controlling the nuclear shuttling of the cytoplasmic PLK1 during the G2-phase and helps explain the observed G2 arrest upon PLK1 dimer stabilization (Fig. 5B, C).

Based on these results, we set out to explore whether the dysregulated nuclear shuttling of the PLK1 dimer has consequences on the subsequent recruitment of PLK1 to its mitotic sublocalizations. To do this, we assessed the mitotic PLK1 localization in the PLK1–3xMyc cells harboring stable PLK1 dimer using immunofluorescence. The analysis showed that the PLK1 dimer stabilization abnormally accumulates PLK1 in the cytoplasm (c-PLK1) of mitotic cells (Fig. 6C, arrowheads, 6D) without affecting the localization of the centromere DNA-bound fraction (d-PLK1). Furthermore, the kinetochore-associated pT210 signal was not altered in the presence of a stable PLK1 dimer (Fig. 6C, E). However, pT210 signals did not co-localize with the displaced PLK1 suggesting that the cytoplasmic secluded PLK1 (most likely dimeric PLK1) is catalytically inactive (Fig. 6C). Together, the results indicate that the PLK1 dimer dissociation during late G2 is essential for the NLS-dependent nuclear translocation of PLK1. Interfering with this timely regulated PLK1 dimer-monomer switch causes cytoplasmic retention of PLK1 during the late G2 phase, thereby contributing to the dysregulation of mitosis entry.

## Discussion

The ability to switch between an active and an inactive state is the key feature of PLK1 that controls the downstream signaling pathways regulating cell division [31]. While the mechanistic aspect of PLK1 activation has been analyzed in detail [28, 41], the questions of when and where this precisely occurs remain elusive [16, 31]. Despite the results of Bruinsma et al., Gheghiani et al., and Seki et al., we were still wondering about the mechanisms regulating the cytoplasmic pool of PLK1. We were intrigued by the fact that cytoplasmic PLK1 is retained in a latent state with unphosphorylated T210 during early G2 despite the local accumulation of its activators, Aur-A and Bora. Hence, the primary aim of our investigation was to decipher the molecular mechanism behind this observation. Here, we propose the transient PLK1 dimerization as a new mechanism regulating the activation of cytoplasmic PLK1 during the G2 phase. Using several independent techniques such as co-IPs, FRET, Nanobit, and biochemical assays like the BS3-crosslinking and SEC, we could demonstrate that during the G2 phase, PLK1 homodimers exist. Additionally, the PLK1 homodimerization is promoted by Bora upon the CDK1-dependent phosphorylation of Bora’s S252 residue. Interestingly, SEC and BS3-crosslinking analyses showed that Bora also exists as a homodimer during the G2 phase, in complex with PLK1 dimers, supporting that Bora dimers sustain PLK1 dimerization during the G2-phase.

Intriguingly, we found that phosphorylation of PLK1-T210 abrogates PLK1 dimerization suggesting the central role of the kinase domain for PLK1 homodimerization and highlighting the role of Aur-A in dissociating PLK1 dimers during late G2. Indeed, the expression of the phosphomimetic mutant PLK1-T210E disrupted the ability of PLK1 to homodimerize and generated monomeric PLK1. Conversely, the inhibition of Aur-A stabilized PLK1 dimers concomitant with a loss of T210 phosphorylation in cells. By implication, this data hinted that the monomeric PLK1 differs from the dimeric PLK1 in terms of their catalytic activity. Time kinetic experiments showed the presence of PLK1 dimers during the G2 phase and exclusively in the cytoplasmic fraction of cells. Moreover, the dissociation of the PLK1 dimer occurred shortly before mitotic entry, meaning that despite being complexed to Bora and Aur-A during the entire G2, the PLK1 dimer remains unphosphorylated on Thr 210.

**Fig. 7:**
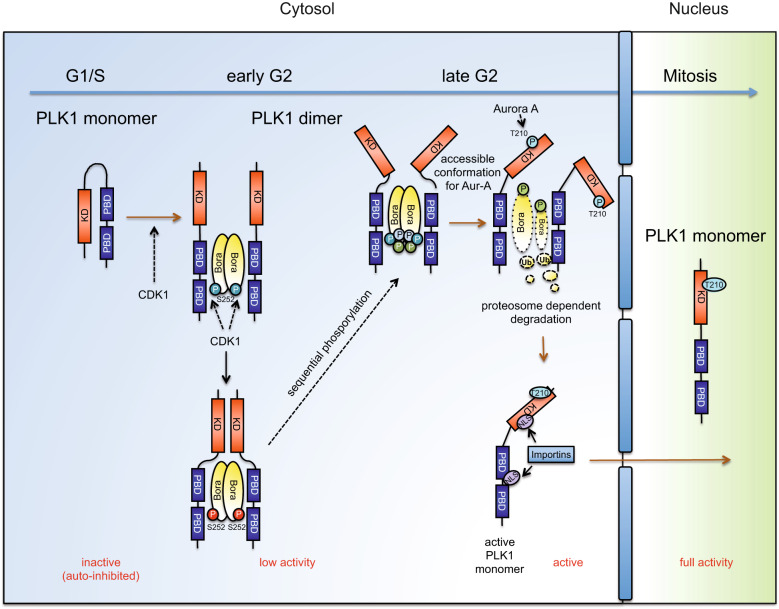
Model for the regulation of PLK1 dimerization. The association of PBD and KD autoinhibits PLK1. The CDK1-dependent priming event of Bora promotes the dimerization of cytoplasmic PLK1 during early G2. The sequential hyperphosphorylation of Bora during G2 might be one mechanism that triggers structural modifications of the PLK1 dimers. These alterations will ultimately generate a dimer conformation where the T-loop within PLK1 becomes accessible for the Aur-A-dependent phosphorylation of T210 in late G2. T210 phosphorylation and the subsequent Bora degradation lead to the dissociation of PLK1 dimer. This dissociation supports the NLS-dependent nuclear translocation of PLK1 through interaction with importins.

These observations are of particular interest, as they are supported by a study where the authors found that PLK1 holds a closed conformation within the PLK1-Aur-A-Bora complex [52]. Additional data indicated that the N-terminal domain of Bora associated with PLK1 undergoes a sequential and timely defined succession of phosphorylation events that change Bora’s conformation, and consequently, generate subtle changes in PLK1 conformation [52]. Accordingly, we are tempted to speculate that these sequential Bora phosphorylation events, which would begin already from early G2, and the PBD dependent association with other PLK1 activating factors such as the WW-domain-containing adaptor protein with a coiled-coil region (WAC) [53], may gradually alter PLK1’s conformation. At late G2, these conformational changes, in addition to dephosphorylation events carried out by the CDK1 antagonizing phosphatases (mostly PP2A-B55), might generate a PLK1 dimer with an accessible T-loop enabling Aur-A phosphorylation of T210 leading to PLK1 activation and the subsequent Bora degradation. These events promote the release of the dimers and the switch to the PLK1 monomer active form, facilitating importin-dependent nuclear entry during early mitosis (Fig. 7). This will ultimately lead to the activation of Cyclin B1/CDK1 and mitotic entry. Importantly, blocking the PLK1 dimer to monomer switch seems to result in dysregulated G2-M transition, mainly due to the altered cascade of signaling events controlled by the axis PLK1-Aur-A-Bora. Hence, our PLK1 dimerization would represent a new mechanism to fine-tune the timely activation of cytoplasmic PLK1 during the late G2 phase.

The recent study of Zhu et al. also observed using crystallography that drosophila’s Polo (PLK1) and, more precisely, the PDB domain of PLK1 could dimerize upon its association with the “adaptor protein partner of Numb” (Pon) [54]. In this context, CDK1 specifically phosphorylated the Thr 63 of Pon, thereby creating a docking site facilitating the binding of Pon with the PLK1-PBD. This interaction induced the dimerization of PLK1-PBD and the full-length protein in solution. However, more intriguingly, while our study reports that the PLK1 dimerization maintains PLK1 in a low active state, Zhou et al. observed that the dimerization stimulates PLK1 enzymatic activity by 3-folds. Thus, discrepancies are existing in the results of both studies that might be ascribed to different reasons:

i) While Zhou’s study reports that the dimerization of PLK1 occurs exclusively through the PLK1-PBD domain upon the association with Pon leaving the KD domain free and accessible for activating phosphorylations by upstream kinases, we describe that both PLK1-PBD and PLK1-KD dimerize within the full-length PLK1. However, after the dimerization of PLK1-KD, PLK1-T210, which phosphorylation is necessary for PLK1 activation, remains inaccessible for Aurora-A, thus keeping the cytoplasmic PLK1 pool in a low active state until shortly before mitosis. ii) Secondly, most of the experiments performed in the Zhou et al. study were in vitro experiments in a cell-free system and mainly using a fusion protein of bacterial PLK1-PBD. More importantly, omitting the intracellular upstream regulation, such as phosphorylation and dephosphorylation events that may be crucial for dimer activation/inactivation, could render the interpretation of the in vitro data somewhat incomplete.

Recently and in an elegant set of experiments, Singh et al. challenged the self-priming model proposing that PLK1 phosphorylates PBIP1/CENP-U at Thr 78 and subsequently docks onto it [55]. They showed that CDK1 activity is necessary for PLK1 recruitment to the kinetochore. Initial phosphorylation by CDK1 of CENP-U Thr 98 will ignite the recruitment of a first PLK1 molecule. After successful phosphorylation of the spatially adjacent Thr 78 on CENP-U, a second PLK1 molecule is recruited. Finally, both PLK1 molecules are stabilized by dimerization, presumably through the PBD domains. However, this model of mitotic PLK1 dimerization appears to differ from our model for PLK1 dimerization in the G2 phase significantly. The mitotic model of PLK1 dimerization described by Singh, P. et al. promotes the stabilization of PLK1 at kinetochores and, most importantly, supports maintaining PLK1 in a very high activation state which is consistent with the various tasks performed by this protein during the different phases of mitosis (Kinetochore-MT Attachments, Spindle assembly checkpoint, anaphase onset). In contrast, the dimerization of PLK1 during early G2 aims to maintain the protein in a low activation state and thus prevent an untimely G2-M transition.

The functions of PLK1 are regulated by multiple post-translational modifications and through its spatial and temporal distribution. Evidence suggests that the nuclear import of PLK1 during G2 is mandatory for the proper mitotic progression, and interfering with this shuttling arrests the cells in the G2 phase. This translocation is mediated by the NLS localized in the kinase and PBD domains [49–51]. This data raised the question of whether the NLS is shielded in the human PLK1 dimer, thereby preventing its nuclear entry. Indeed, the stabilization of dimer PLK1 in the G2 phase significantly reduced the association of PLK1 with importin and consequently decreased the amount of nuclear PLK1 required to sustain mitotic entry. Furthermore, interfering with the nuclear import of PLK1 caused by the stabilized dimer resulted in mitotic consequences for PLK1. Cells that could enter mitosis despite the stabilized PLK1 dimer showed aberrant cytoplasmic accumulation of PLK1, indicating that preventing PLK1 dimer-to-monomer switch during the G2 phase, and thus interfering with its nuclear transport, hinders PLK1 recruitment to mitotic subcellular localizations.

Reactive oxygen species (ROS) have been considered a side product of oxygen metabolism for a long time and are now considered an important modulator of cell signaling pathways [56]. ROS levels progress through the cell cycle with a peak in mitosis, suggesting the relevance of the redox-modulation on the regulation of mitotic kinases. Indeed, during mitosis, Aur-A undergoes the oxidation of the Cysteine 290 (C290), which lies adjacent to the T288, the critical phosphorylation site, within the activation loop [57, 58]. According to Lim et al., the CoAlation of Aur-A-C290 would promote its dimerization and autophosphorylation [59]. Intriguingly, C290 equivalents are highly conserved in a cohort of human protein kinases like the PLK family. Mutation of PLK1-C172 abolished PLK1 activity, indicating this residue’s role in regulating the catalytic activity of PLK1 [57]. Our study cannot rule out that the redox-dependent regulation of PLK1-C172 influences the dimerization of PLK1, comparable to Aur-A, and consequently regulates its activity during the G2 phase. Additional work is required to provide more insights into this matter.

Volasertib (BI6727) is the most advanced PLK1 inhibitor explored clinically [60, 61]. However, the adverse events caused by its limited specificity might suggest the need for improvement as a therapeutic agent. In this regard, our study proposes Bora as an allosteric modulator regulating the dynamics between dimeric and monomeric active PLK1. PLK1-Bora interaction might help to establish a co-crystal structure, which will lay the ground for designing a new generation of allosteric compounds mimicking these interactions and thus inhibiting the PLK1 downstream signaling pathways by stabilizing or preventing the dimeric and inactive conformation. Additional supporting data for the efficacy of allosteric PLK1 inhibitors arises from our recent work. We showed that compounds affecting the conformation of PLK1 disrupt its association with interacting partners and proved to be effective inhibitors of PLK1 signaling, despite showing a very weak efficiency in inhibiting its catalytic activity in vitro [62]. However, as a first step, as the data of Bora-PLK1 co-crystal structure is still not yet available, a screening of already existing libraries of compounds for their possible interaction with PLK1 may help discover putative allosteric regulators for PLK1. Finally, we need to reconsider and study the existing PLK1 inhibitors currently in clinical trials for their ability to affect PLK1 dimer-monomer switch in cells as this mechanism turns out to be important for the regulation of PLK1 activity. The data gathered may then be used to improve the already existing spectrum of PLK1 inhibitors by designing a new generation of small molecule inhibitory drugs targeting PLK1 for clinical cancer trials.

## Supplementary information

Supplemental Figure 1

Supplemental Figure 2.1

Supplemental Figure 2.2

Supplemental Figure 3

Supplemental Figure 4

Supplemental Figure 5

Supplemental Information
