# Correction: A dimerization-dependent mechanism regulates enzymatic activation and nuclear entry of PLK1

**DOI:** 10.1038/s41388-023-02779-3

**Published:** 2023-07-24

**Authors:** Monika Raab, Yves Matthess, Christopher A. Raab, Niklas Gutfreund, Volker Dötsch, Sven Becker, Mourad Sanhaji, Klaus Strebhardt

**Affiliations:** 1 Department of Gynecology, Medical School, Goethe University, Frankfurt, Germany; 2 Institute of Biophysical Chemistry and Center for Biomolecular Magnetic Resonance, Goethe University, Max-von-Laue Str. 9, 60438 Frankfurt am Main, Germany; 3 German Cancer Consortium (DKTK) / German Cancer Research Center, Heidelberg, Germany

**Keywords:** Mitosis, Cell division

Correction to: *Oncogene* 10.1038/s41388-021-02094-9, published online 10 November 2021

In this article the statement in the Funding information section was incorrectly given as ‘This work was supported by grants from the Deutsche Forschungsgemeinschaft (M. Raab), Messer Stiftung, Deutsche Krebshilfe, German Cancer Consortium (DKTK), Heidelberg and Verein zur Förderung der Wissenschaft, Prävention and Therapie von Kehlkopfkrebs e.V. Open Access funding enabled and organized by Projekt DEAL.’ and should have read

‘This work was supported by grants from the Sander Stiftung (Nr. 2021.023.1), Deutsche Krebshilfe (70114007), German Cancer Consortium (DKTK), Heidelberg. Open Access funding enabled and organized by Projekt DEAL.’

The original article has been corrected.

