# Supplementary figures and images for: A dimerization-dependent mechanism regulates enzymatic activation and nuclear entry of PLK1
Source: Oncogene. 2021 Nov 10;41(3):372–86. doi: 10.1038/s41388-021-02094-9 (PMC8755526; doi:10.1038/s41388-021-02094-9)

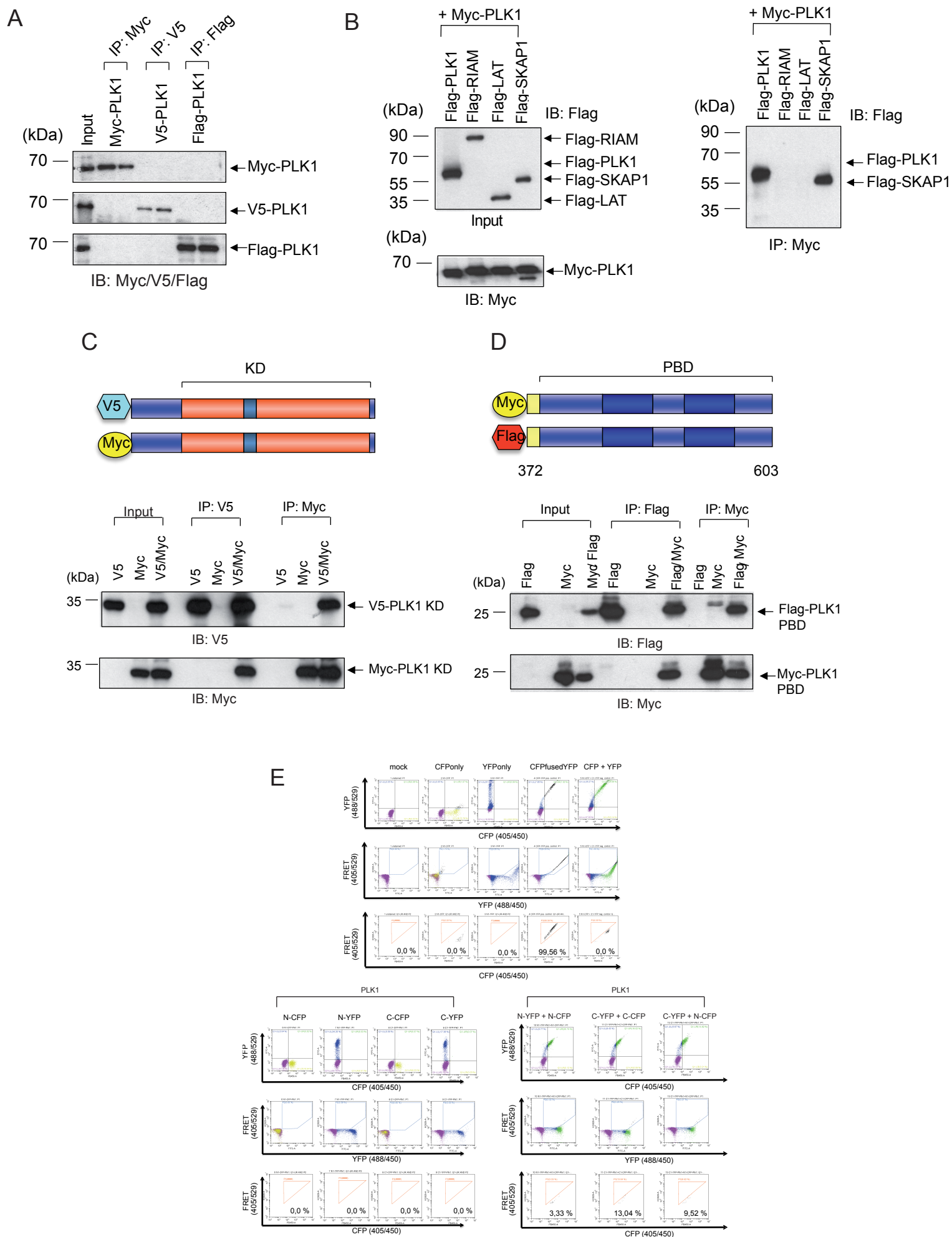

Figure S1.

Supplement: Supplementary file 1 — Supplemental Figure 1 [file 41388_2021_2094_MOESM1_ESM.pdf]

A

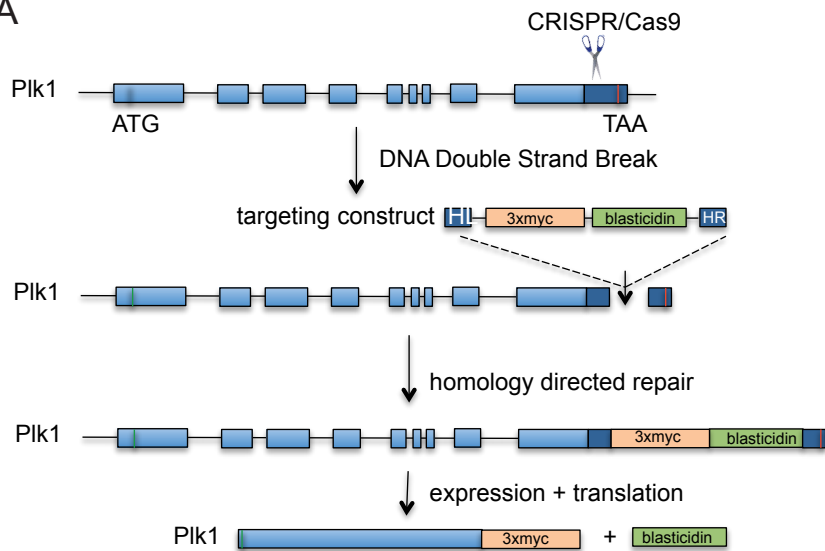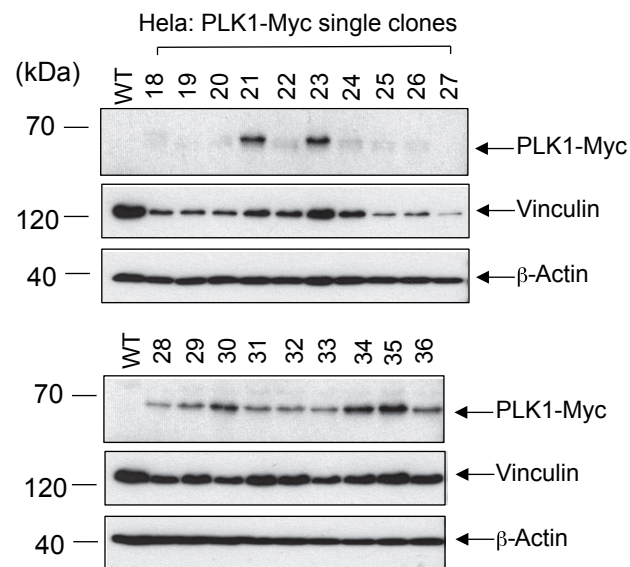

B

Myc-PLK1 KD + V5-PLK1 KD

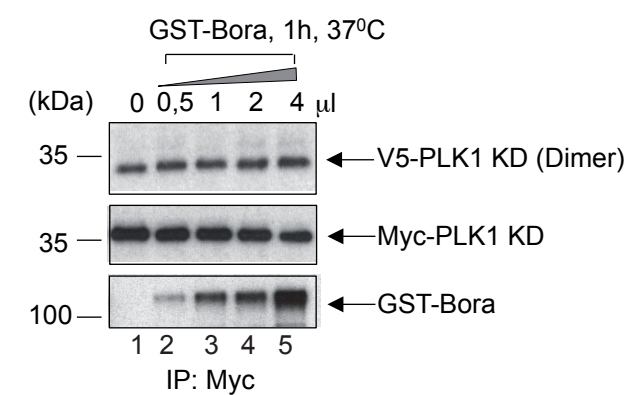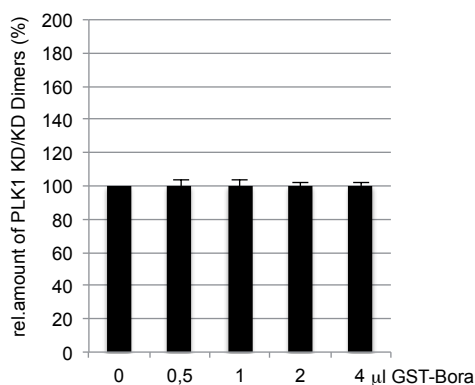

C

Myc-PLK1 PBD + V5-PLK1 PBD

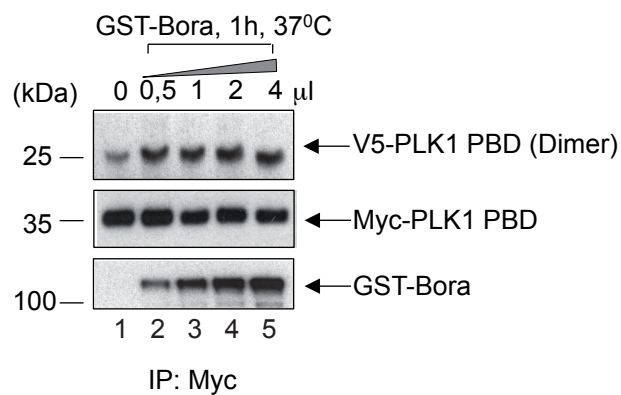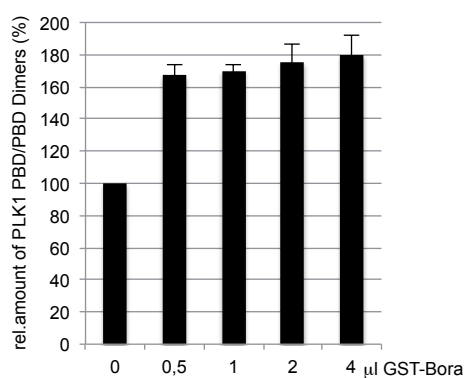

D

V5-PLK1 PBD + Myc-PLK1 PBD

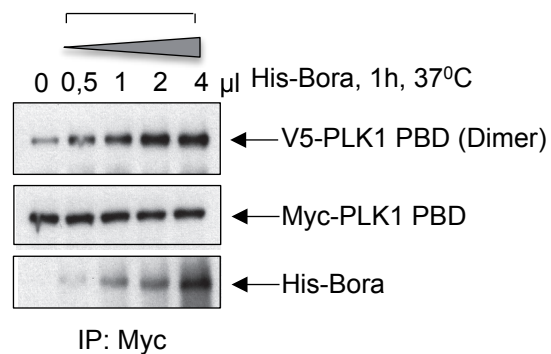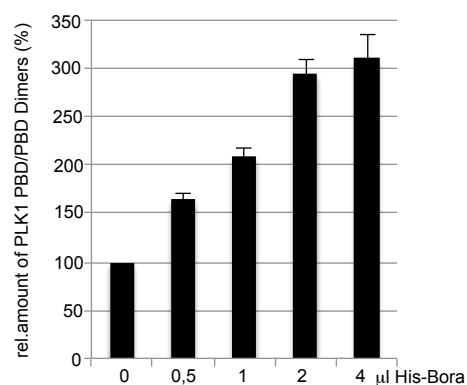

Figure S2.

Supplement: Supplementary file 2 — Supplemental Figure 2.1 [file 41388_2021_2094_MOESM2_ESM.pdf]

E

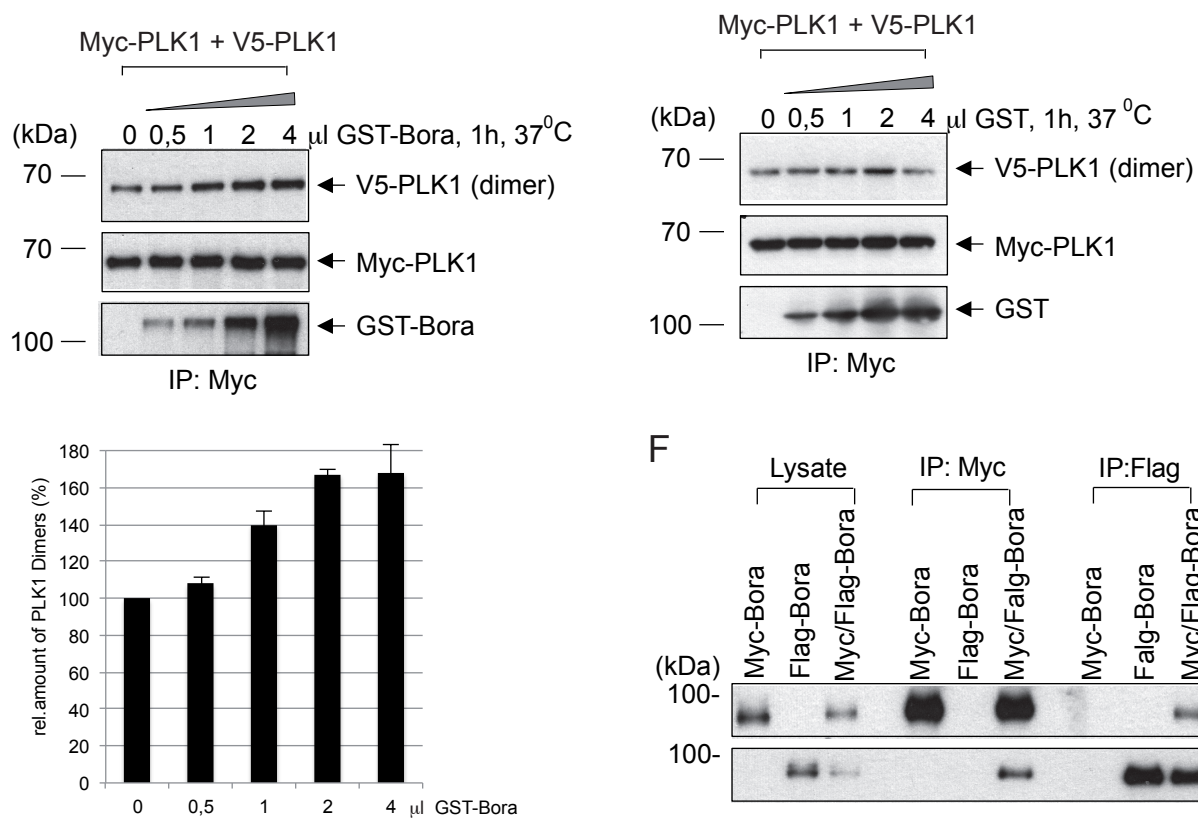

F

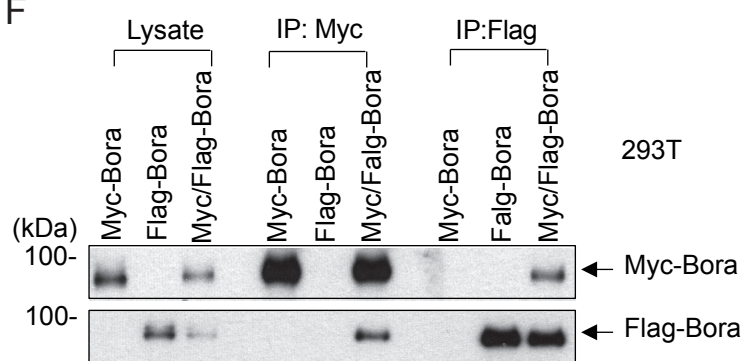

G

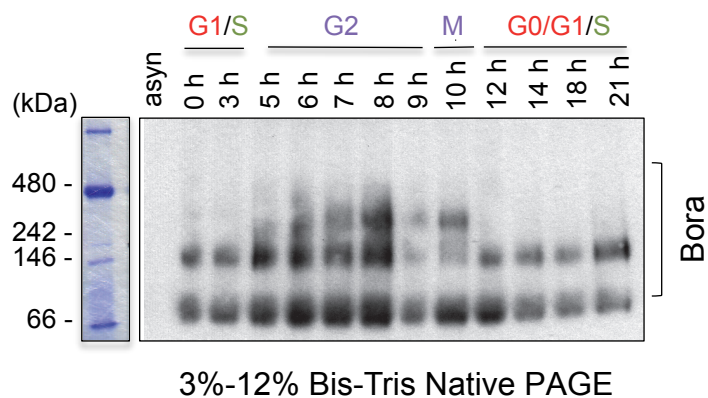

Figure S2.

Supplement: Supplementary file 3 — Supplemental Figure 2.2 [file 41388_2021_2094_MOESM3_ESM.pdf]

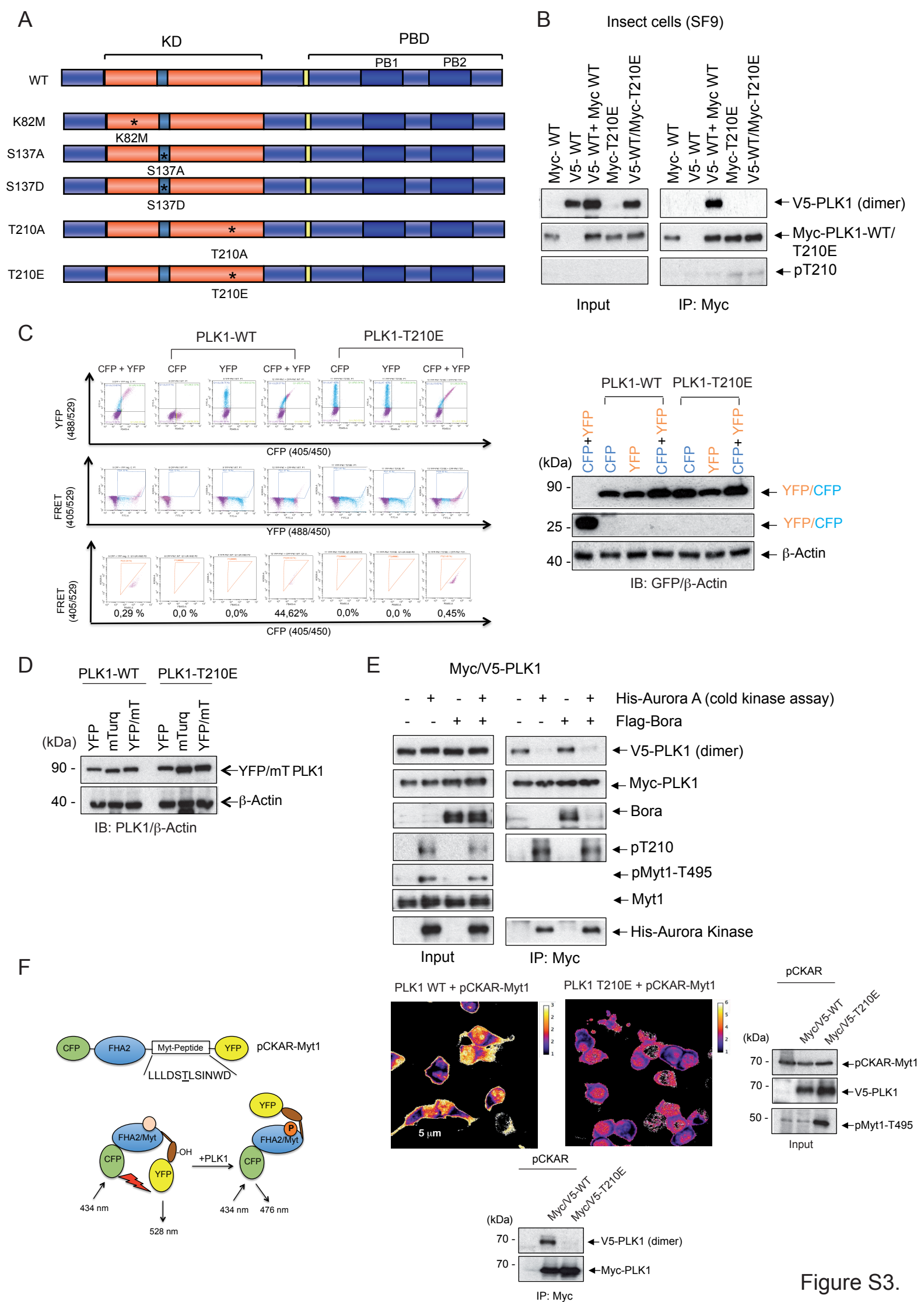

Supplement: Supplementary file 4 — Supplemental Figure 3 [file 41388_2021_2094_MOESM4_ESM.pdf]

A

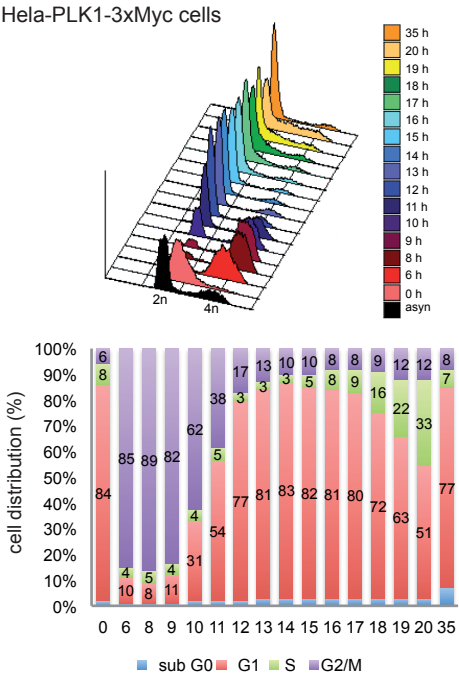

B

Hela WT

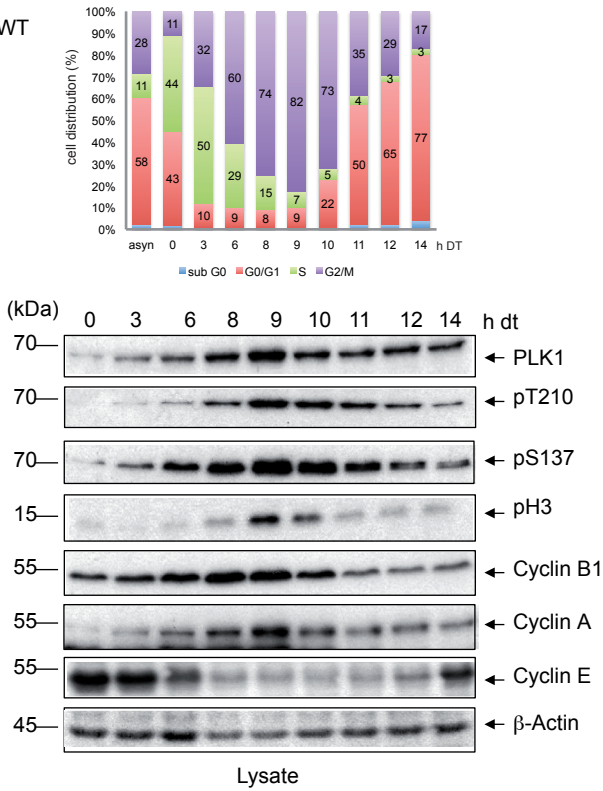

Figure S4.

Supplement: Supplementary file 5 — Supplemental Figure 4 [file 41388_2021_2094_MOESM5_ESM.pdf]

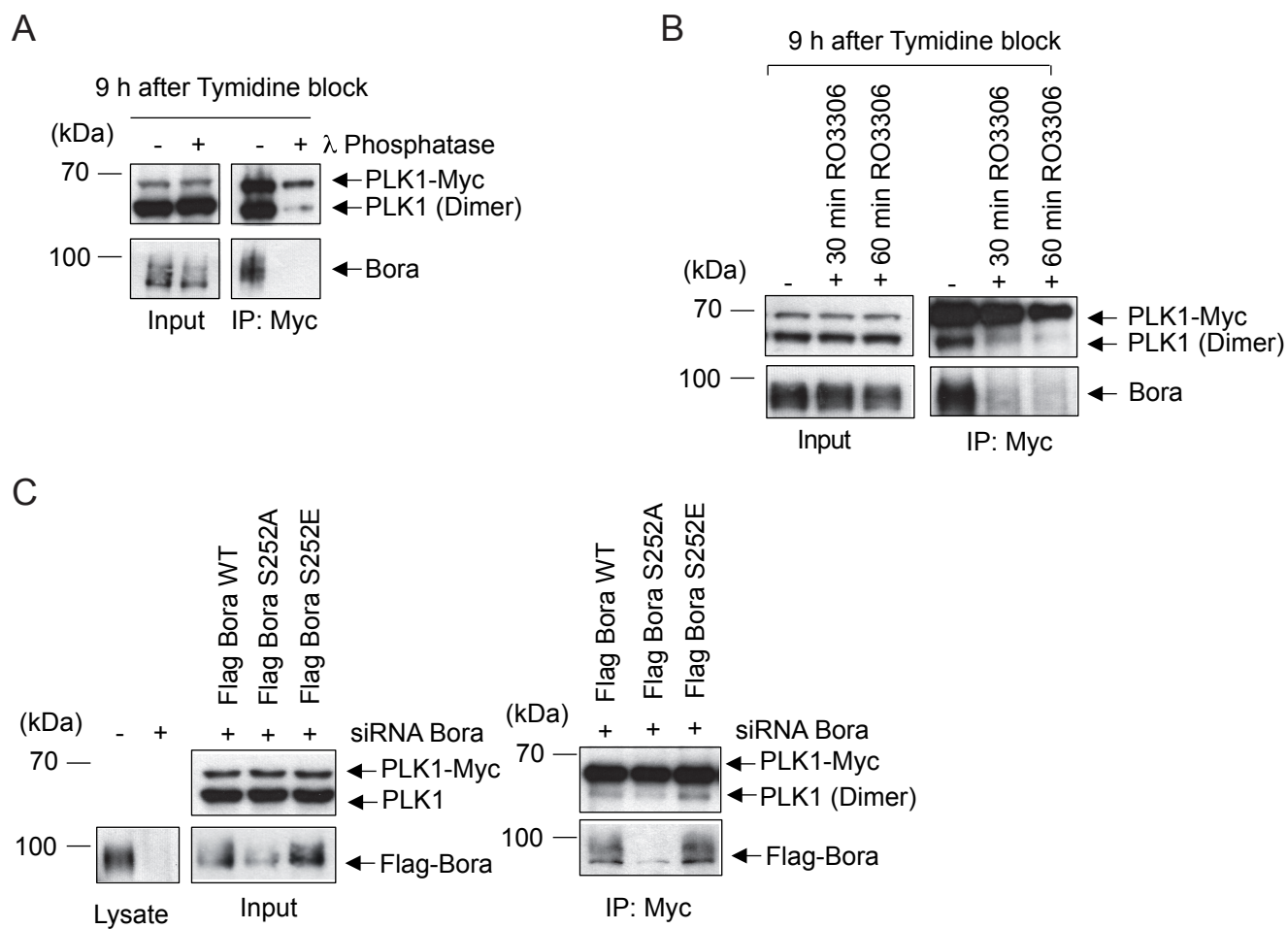

Figure S5.

Supplement: Supplementary file 6 — Supplemental Figure 5 [file 41388_2021_2094_MOESM6_ESM.pdf]
