# Supplementary material for: A dimerization-dependent mechanism regulates enzymatic activation and nuclear entry of PLK1
Source: Oncogene. 2021 Nov 10;41(3):372–86. doi: 10.1038/s41388-021-02094-9 (PMC8755526; doi:10.1038/s41388-021-02094-9)
Supplement: Supplementary file 7 — Supplemental Information [file 41388_2021_2094_MOESM7_ESM.docx]

**A dimerization-dependent mechanism regulates enzymatic activation**

**and nuclear entry of PLK1**

Monika Raab^1^, Yves Matthess^1^, Christopher A. Raab^1^, Niklas Gutfreund^2^, Volker Dötsch^2^, Sven Becker^1^, Mourad Sanhaji^1*†^, Klaus Strebhardt^1,3*†^

*corresponding authors: Klaus Strebhardt and Mourad Sanhaji

Klaus Strebhardt: [strebhardt@em.uni-frankfurt.de](mailto:strebhardt@em.uni-frankfurt.de)
Mourad Sanhaji: mourad.sanhaji@kgu.de

**Supplementary Information**

**Supplemental Figures legends**

**Fig. S1. PLK1 dimerization in vivo and in vitro.**

(**A)**Control for antibody specificity. HEK293T cells were transfected with Myc-, V5- or Flag-PLK1. Myc-, V5- or Flag-PLK1 were immunopurified from cell lysates. The indicated proteins were analyzed by western blotting. (**B)**(Upper) Cells were co-transfected with Flag-PLK1, -RIAM, -LAT, -SKAP1, and Myc-PLK1. (Right) Myc-PLK1 was immunopurified from cell lysates. The indicated proteins were analyzed by western blotting. (**C-D)** (Upper) Schematic representation of the kinase domain (KD), and the polo-box domain (PBD). (**C)** Lysates of HEK293T cells expressing Myc- and V5-tagged KD were subjected to immunoprecipitation (IP) using V5-, or Myc-specific antibodies and western blotting using V5- or Myc-antibodies. (**D)** Lysates of HEK293T cells co-expressing the Myc- and Flag-tagged PBD were subjected to immunoprecipitation (IP) using Flag- or Myc-specific antibodies and western blotting using Flag- or Myc-antibodies. (**E)** FRET measurements by flow cytometry. Representative primary FACS-plots showing FRET+ cells in a population of living HEK293T cells co-transfected with the indicated CFP/YFP fusion proteins and analyzed by CytoFlex flow cytometer. Numbers give the total percentage of cells within the FRET gate. Double positive cells were gated (upper). False-positive signals from YFP being excited at 405 nm were excluded by adding an additional gate (middle). In the bottom panel, the gate was adjusted to exclude cells transfected with CFP or YFP only and are thus FRET negative.

**Fig. S2. Bora promotes the oligomerization of the PLK1 PBD**

**(A)**CRISPR/Cas9-based tagging of endogenous Plk1. (Left) Schematic presentation of the knock-in strategy by CRISPR/Cas9 for the fusion of the Plk1 locus to a 3xMyc-tag. (Right) Analysis of CRISPR/Cas9-generated single clones by western blotting with antibodies for PLK1-Myc, Vinculin, and β-Actin as loading controls.**(B, C)**Bora promotes the dimerization of the PLK1 PBD: Increasing amounts of purified GST-Bora were added to lysates of HEK293T cells expressing **(B)** Myc- and V5-PLK1 KD or **(C)** Myc- and V5-PLK1 PBD. Subsequently, a co-IP using anti-Myc was performed. The relative amount of **(A)**KD or **(B)** PBD dimers were determined and quantified. **(D) (Left)** Lysates of HEK293T cells expressing Myc, V5-tagged PBD were incubated with increasing amounts of purified His-Bora. Subsequently, a co-IP with anti-Myc was performed and western blotting using V5- or Myc-antibodies and His-antibodies has been carried out. (Right) The relative amount of PBD dimers were determined and quantified  **(E)**Increasing amounts of purified GST-Bora (left) or GST (right) were added to HEK293T cell lysates expressing Myc- and V5-PLK1 full-length protein. Subsequently, a co-IP using anti-Myc was performed. The relative amount of PLK1 dimers were determined and quantified. **(F)** Cell lysates of HEK293T expressing either Flag-tagged or Myc-tagged Bora or both were subjected to (IP) using Flag-,Myc-specific antibodies followed by western blotting using Flag- or Myc-antibodies. **(G)**Hela cells were synchronized to G1/S boundaries by a thymidine treatment and release for the indicated time points. The synchronized cells were lysed separated by Blue NATIVE PAGE and subjected to western blotting using anti-Bora antibody.

**Fig. S3. The T210 phospho-mimicking mutation blocks PLK1 dimerization**

(**A)** Domain architecture of PLK1 and location of functionally relevant residues (K82, S137, T210). (**B)** SF9 cells were transfected with Myc-, V5-PLK1-WT or Myc-PLK1-T210E or combinations as indicated. Myc-PLK1 was immunopurified from cell lysates. Myc-PLK1 and the co-IPed proteins were analyzed by western blotting. (**C)** (Left) Representative primary FACS plots showing FRET+ cells within a population of living HEK293T co-transfected with the indicated CFP and YFP fusion proteins and analyzed by CytoFlex flow cytometer. (Right) HEK293T cells were co-transfected with C-terminal CFP/YFP-fused PLK1 WT and -PLK1-T210E constructs alone or in combination and 24 h later blotted with anti-GFP and β-Actin. **(D)** Lysates of HEK293T cells expressing the YFP and mTurquoise constructs of PLK1-WT and PLK1-T210E were blotted with anti-PLK1 and anti-β-Actin. **(E)**HEK293T cells were transfected with Myc-, V5-PLK1, and Flag-Bora. The cell lysates were incubated with purified His-Aur-A in a cold kinase assay. Subsequently, the phosphorylated lysates were subjected to a Myc-IP, and the indicated proteins were analyzed by western blotting. **(F)**(left) Schematic presentation of a FRET-based biosensor for PLK1 activity in live cells. (middle upper panel) Quantifications of CFP/YFP emission ratios of cells expressing PLK1-WT or PLK1-T210E and pCKARMyt1. (Middle lower panel) Cells were subjected to co-IP using anti-Myc, and precipitates were immunoblotted for V5 and Myc. (Right) Cells were immunoblotted for pCKAR-Myt1, V5-PLK1, and Myt1-pT495.

**Fig. S4. Regulation of PLK1 in space and time during the cell cycle**

(**A)** Regulated dimerization of endogenous PLK1 during the cell cycle. HeLa-PLK1-3xMyc cells were synchronized at the G1/S boundary by a double thymidine arrest (dt), released into fresh medium, and harvested at the indicated times. Cell-cycle stages were determined using fluorescence-activated cell sorting (FACS). (**B)** (Upper panel) (The cell cycle distribution of synchronized HeLa cells represented as a bar graph. (Lower panels) Cell cycle kinetics of HeLa wild-type cells. HeLa cells were synchronized at the G1/S boundary by a double thymidine arrest (dt), released into fresh medium, and harvested at the indicated times. (Upper) Cell-cycle stages were determined using FACS. (Lower) Levels of indicated proteins were analyzed by western blotting.

**Fig. S5. Stabilization of PLK1 dimers by the S252E phospho-mimic mutant of Bora**

**(A)**HeLa-PLK1-3xMyc cells were synchronized by dt treatment and released for 9 h. Lysates were treated with γ-phosphatase. Myc-IP was performed and immunoblotted for PLK1 and Bora. (**B)** HeLa-PLK1-3xMyc cells were synchronized by dt treatment and released for 9 h, followed by incubation for 30 or 60 min with the CDK1 inhibitor RO3306 (9 µM). Lysates were immunoblotted for PLK1 and Bora. The Myc-IP was performed and immunoblotted for PLK1 and Bora. (**C)**HeLa-PLK1-3xMyc cells were transfected for 24 h with siRNA Bora and rescued by transfection with Flag-Bora WT, Flag-Bora S252A, or Flag-Bora S252E mutants. Lysates (left panel) and anti-Myc immunoprecipitates (right panel) were blotted for PLK1 and Bora.
